# The novel antifungal agent NPD2560 perturbs the Rho1-centered signaling network to induce a cell wall integrity response

**DOI:** 10.1128/spectrum.03630-25

**Published:** 2026-06-03

**Authors:** Wei Liu, Hiroki Sekiguchi, Sheena C. Li, Luis Alberto Vega Isuhuaylas, Daisuke Yamanaka, Kaori Itto-Nakama, Yoichi Noda, Charles Boone, Yoko Yashiroda, Yoshikazu Ohya

**Affiliations:** 1 Department of Integrated Biosciences, Graduate School of Frontier Sciences, The University of Tokyo 196861, Kashiwa, Chiba, Japan; 2 RIKEN Center for Sustainable Resource Science 98319, Wako, Saitama, Japan; 3 Terrence Donnelly Centre for Cellular and Biomolecular Research, University of Toronto 7938, Toronto, Ontario, Canada; 4 Laboratory for Immunopharmacology of Microbial Products, School of Pharmacy, Tokyo University of Pharmacy and Life Sciences 146935, Hachioji, Tokyo, Japan; 5 Graduate School of Agricultural and Life Sciences, The University of Tokyo 13143, Bunkyo-ku, Tokyo, Japan; 6 Collaborative Research Institute for Innovative Microbiology, The University of Tokyo 595461, Bunkyo-ku, Tokyo, Japan; 7 Department of Science and Technology Innovation, Nagaoka University of Technology 52756, Nagaoka, Niigata, Japan; Stony Brook University, Stony Brook, New York, USA

**Keywords:** antifungal agents, *Candida krusei*, cell wall integrity pathway, Rho1, Fks1, β-(1,6)-glucan

## Abstract

Fungal pathogens pose an increasing threat to human health. The limited diversity of antifungal drug classes and the rapid emergence of resistance highlight the urgent need for the identification and development of agents with novel mechanisms of action. Here, we identified NPD2560, a coumarin-derived compound, as an antifungal agent that exhibits fungicidal activity against *Candida krusei,* a clinically challenging non-albicans *Candida* species often associated with treatment failure. Chemical-genomic profiling in *Saccharomyces cerevisiae* identified *RHO1* as a major genetic determinant of NPD2560 sensitivity, suggesting perturbation of the Rho1–Fks1 signaling network that regulates fungal cell wall biogenesis and integrity. Mutational analyses revealed that *fks1*Δ and *rho1-3* mutants were hypersensitive to NPD2560, indicating that intact function of this pathway is critical for cellular tolerance to the compound. Biochemical analysis further demonstrated that NPD2560 was associated with a reduction in β-(1,6)-glucan levels, while β-(1,3)-glucan levels were largely unaffected under the conditions tested, leading to activation of the cell wall integrity (CWI) pathway and compensatory chitin deposition. Consistent with these findings, *kre6*Δ and *KRE6*(F552I) mutants defective in β-(1,6)-glucan synthase activity showed no cross-resistance, indicating a mechanism distinct from the β-(1,6)-glucan synthase inhibitor jervine, but functionally linked to the Rho1–Fks1 branch. Transcriptomic analysis revealed a robust remodeling response with induction of Rlm1-dependent cell wall genes such as *CRH1*, *KDX1*, *CWP1*, and *PIR3*. Taken together, these results show that NPD2560 perturbs the Rho1-centered cell wall regulatory network, triggering compensatory cell wall stress responses, and provide a mechanistically distinct chemical probe for dissecting fungal cell wall integrity pathways.

**IMPORTANCE:**

The fungal cell wall is an essential structure that determines cell shape, viability, and pathogenicity, representing an ideal therapeutic focus for antifungal intervention. However, most existing antifungal drugs act on ergosterol or β-(1,3)-glucan synthesis, and resistance among non-albicans *Candida* species remains a serious issue. In this study, we characterized NPD2560, a coumarin-derived compound that functionally perturbs the Rho1–Fks1 signaling pathway involved in cell wall construction. Unlike previously reported coumarin derivatives, which mainly act through oxidative or mitochondrial stress, NPD2560 induces cell wall stress and activates the CWI pathway. These findings provide new mechanistic insights into fungal cell wall control and position NPD2560 as a mechanistic probe and potential lead compound for exploring the cell wall regulatory network.

## INTRODUCTION

Fungal pathogens represent an increasingly serious threat to both global agriculture and human health. In agriculture, fungal pathogens such as *Magnaporthe oryzae*, *Fusarium*, and *Botrytis* species cause major crop losses and postharvest spoilage, posing major challenges to food security and safety (1). It is estimated that fungal diseases destroy between 10% and 23% of global crop production each year—enough to feed over 600 million people—and cause economic losses exceeding 60 billion USD annually (2, 3). These agricultural impacts illustrate the profound consequences of fungal pathogens on the global economy and food supply.

Different groups of pathogenic fungi are responsible for human disease. The major human fungal pathogens include *Candida*, *Aspergillus*, *Cryptococcus*, and *Pneumocystis* species (4). In humans, infections caused by these fungi result in substantial morbidity and mortality, particularly among immunocompromised individuals such as those undergoing chemotherapy, organ transplantation, or living with HIV/AIDS. Recent global estimates suggest that more than 6.5 million cases of severe fungal disease occur each year, leading to approximately 3.8 million deaths (5). Among these, invasive candidiasis accounts for roughly 1.6 million cases annually, with mortality rates exceeding 40% in severe cases despite antifungal treatment (6, 7). Taken together, these observations highlight the enormous global impact of human pathogenic fungi and underscore the urgent need for improved antifungal strategies.

Some fungal pathogens are intrinsically less susceptible to existing antifungal strategies, limiting treatment options. *Candida krusei*, one of the non-albicans *Candida* (NCAC) species, is characterized by intrinsic resistance to fluconazole, reduced susceptibility to other antifungals, and its ability to cause bloodstream infections and hospital outbreaks. Currently, only a few major classes of antifungal drugs are available, including echinocandins (e.g., caspofungin), which inhibit β-(1,3)-glucan synthesis in the cell wall; polyenes (e.g., amphotericin B), which disrupt fungal membranes by binding to ergosterol; azoles (e.g., fluconazole), which inhibit ergosterol biosynthesis; and nucleic acid synthesis inhibitors such as flucytosine (6, 7). Although these agents have significantly improved clinical outcomes, currently available antifungal drugs are limited by toxicity, narrow spectra of activity, and, in some cases, fungistatic rather than fungicidal effects, depending on the drug class and fungal species. As the number of available antifungal classes is limited, resistance can develop rapidly, reducing treatment options and underscoring the urgent need for agents with novel mechanisms of action.

The fungal cell wall is an indispensable and highly conserved extracellular matrix that maintains cellular integrity and morphology (8). Because this structure is absent in mammalian cells, it represents an attractive and selective target for antifungal interventions. Clinically, echinocandins such as caspofungin, micafungin, and anidulafungin inhibit β-(1,3)-glucan synthase, thereby compromising the cell wall integrity (CWI) and leading to growth inhibition and, in some species, cell lysis (6, 8). Echinocandins are widely used as first-line therapies for invasive *Candida* infections, whereas for *Aspergillus* infections, azoles such as voriconazole remain the recommended first-line treatment. Although echinocandins are generally well tolerated, their clinical utility is constrained by the spectrum of activity, cost, and the emergence of resistant strains. Beyond the clinic, several additional agents that interfere with cell wall biosynthesis have been identified experimentally: nikkomycin Z inhibits chitin synthase (9), D75-4590 and jervine block β-(1,6)-glucan formation (10, 11), and poacic acid perturbs β-(1,3)-glucan assembly (12). More recently, the Rho1/RhoA GTPase inhibitor O1 was shown to impair upstream signaling within the cell wall integrity (CWI) pathway (13). Although these compounds have not yet been approved for clinical use, they broaden the mechanistic landscape of cell wall-targeting agents and may guide the design of safer and more durable antifungal therapies.

The budding yeast *Saccharomyces cerevisiae* is a well-established model organism for antifungal research due to its fully annotated genome, advanced genetic toolbox, and strong conservation of fundamental cellular processes with pathogenic fungi. In particular, the architecture and composition of its cell wall, primarily β-(1,3)- and β-(1,6)-glucans, chitin, and mannoproteins, closely mirror those of pathogenic yeasts such as *Candida* species, providing a relevant and experimentally tractable system for studying cell wall biosynthesis and antifungal drug responses (14, 15). Due to its non-pathogenic nature, rapid growth, and extensive genomic resources, *S. cerevisiae* enables large-scale chemical-genomic screening, target identification, and resistance profiling using knockout and overexpression libraries (16, 17). This experimental flexibility has made *S. cerevisiae* a cornerstone for the discovery of novel antifungal mechanisms and for elucidating conserved cellular pathways underpinning fungal viability and drug susceptibility. Although *S. cerevisiae* has long served as a powerful model for studying fungal biology, its potential for systematic discovery of antifungal targets has not yet been fully realized, largely because previous approaches have been limited in scale and pathway coverage.

To identify new antifungal agents that affect fungal cell wall integrity through previously uncharacterized mechanisms, we performed genome-wide chemical-genomic screening of more than 13,000 compounds, including natural compounds, using the model yeast *S. cerevisiae* (18). Candidate compounds showing growth inhibition and hypersensitivity in cell wall-related mutants (18) were prioritized for further analysis. Among these, the coumarin derivative NPD2560 emerged as a compound with reproducible antifungal activity and exhibited measurable activity against NCAC species, including *C. krusei*, the only NCAC species that showed a detectable MIC in our assays. Given its distinctive antifungal profile and previously unexplored mechanism, we initiated an investigation into the cellular processes underlying the action of NPD2560. Subsequent functional assays and transcriptomic profiling revealed that NPD2560 perturbs the Rho1-centered signaling network and is associated with reduced β-(1,6)-glucan levels, thereby activating the CWI pathway. Taken together, these findings position NPD2560 as a mechanistically distinct antifungal compound that perturbs Rho1-dependent cell wall regulatory processes, differing from existing antifungal drugs.

## RESULTS

### NPD2560 exhibits antifungal activity against *Candida krusei*

**Fig 1:**
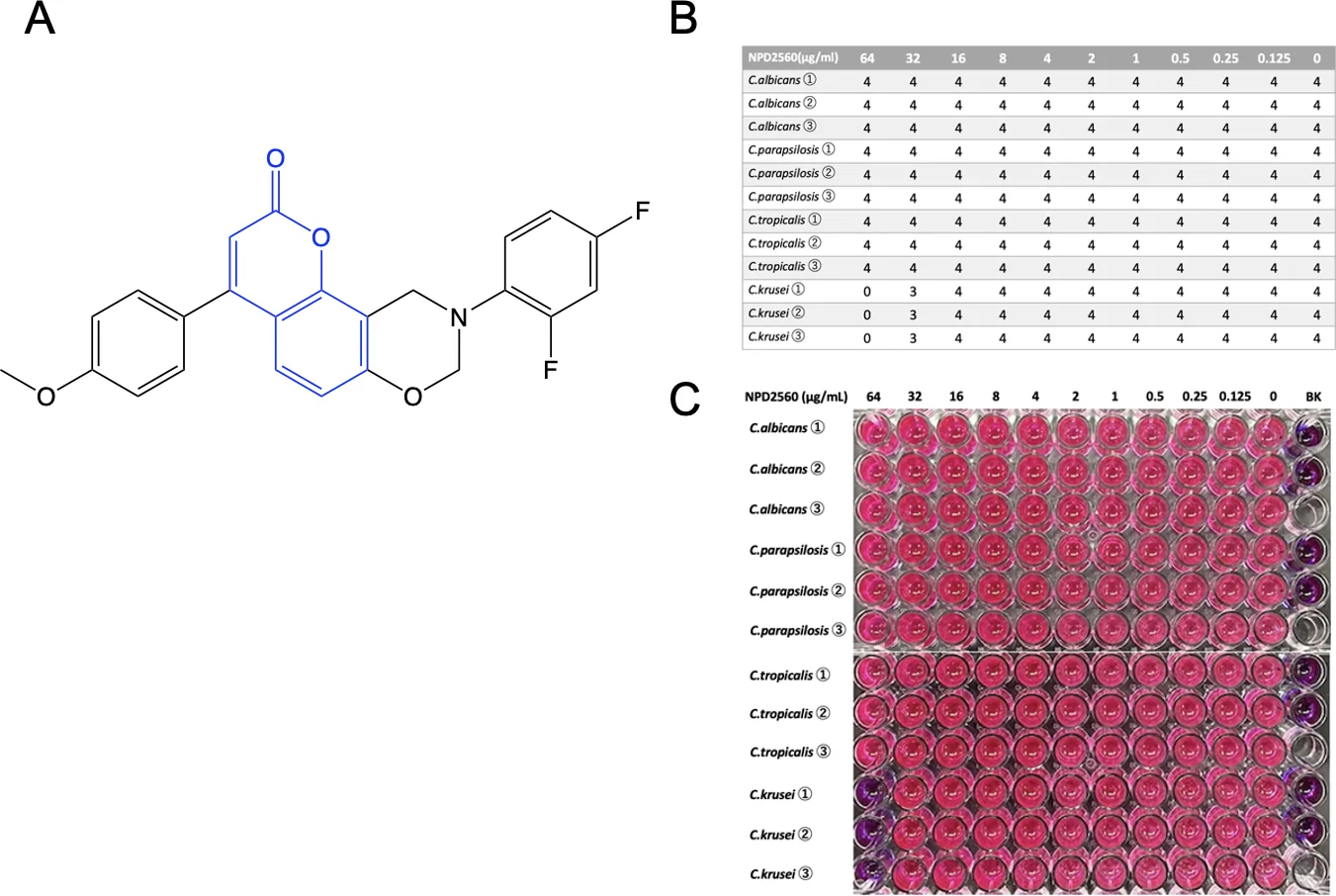
Antifungal activity of NPD2560 against *Candida krusei*. (**A**) Chemical structure of NPD2560. (**B**) Colony count assay. Growth inhibition of *C. krusei* was assessed by colony counting. Colony formation was scored on a scale of 0–4, where 0 indicates complete inhibition (no colonies) and 4 represents normal growth in the absence of the test drug; intermediate values reflect partial inhibition at the corresponding concentrations. (**C**) Resazurin cell viability assay. Metabolic activity was measured using resazurin, which is reduced from blue/purple to pink resorufin in viable cells. Final NPD2560 concentrations tested were 0, 0.125, 0.25, 0.5, 1, 2, 4, 8, 16, 32, and 64 μg/mL.

Yeast deletion mutants of cell wall-associated genes, including *chs7*Δ, *skt5*Δ, *pfa4*Δ, *rgd1*Δ, and *rom2*Δ, were reported previously to show hypersensitivity to the compound investigated in this study, NPD2560 (Fig. 1A) (18). These findings suggested that NPD2560 may exert its antifungal effects by perturbing pathways involved in fungal cell wall biogenesis. To assess the antifungal activity of NPD2560 against clinically relevant human pathogens, we examined its effects against *Candida albicans*, *Candida parapsilosis*, *Candida tropicalis*, and *C. krusei*, all of which are major causative agents of invasive candidiasis (19). Colony-forming unit (CFU) analysis revealed that NPD2560 inhibited the growth of *C. krusei* by approximately 25% at 32 μg/mL and completely suppressed colony formation at 64 μg/mL, indicating fungicidal activity (Fig. 1B). These findings were further supported by a resazurin-based viability assay (20). In this assay, the purple oxidation–reduction indicator resazurin is reduced to resorufin, which is pink, in the presence of metabolically active cells; a purple color therefore indicates loss of viability (20). NPD2560 was shown to inhibit the growth of *C. krusei* at 32–64 μg/mL and completely suppressed colony formation at 64 μg/mL, indicating measurable fungicidal activity (Fig. 1C). This inhibitory profile was comparable to that of fluconazole (IC_90_ = 32 µg/mL), indicating that NPD2560 exhibits a similar level of antifungal activity against *C. krusei*, a species intrinsically resistant to azoles (Table 1).

**TABLE 1:** Antifungal activities of antifungal agents (CLSI method)

Strain	Value for^*a*^			
	NPD2560		FLC	
	MIC_90_	MIC_50_	MIC_90_	MIC_50_
*Candida albicans* ATCC 24433	>64	>64	4	0.25
*Candida parapsilosis* ATCC 22019	>64	>64	2–8	1
*Candida tropicalis* ATCC 750	>64	>64	16	4
*Candida krusei* ATCC 6258	32–64	32–64	>32^*b*^	16–32

^*a*^ MIC was tested using RPMI as the medium by the CLSI (formerly NCCLS) M27-A2 method (21) and was determined after 24 or 48 h of incubation. MIC_50_ was determined as a prominent decrease in turbidity compared with a drug-free control, and MIC_90_ was determined as the lowest drug concentration supporting no visible growth after 24 or 48 h of incubation. FLC, fluconazole.

^*b*^ The quality control was verified according to the criteria described in CLSI M60 (22).

### Chemical-genomic profiling identified *RHO1* as a major genetic determinant of NPD2560 sensitivity

**Fig 2:**
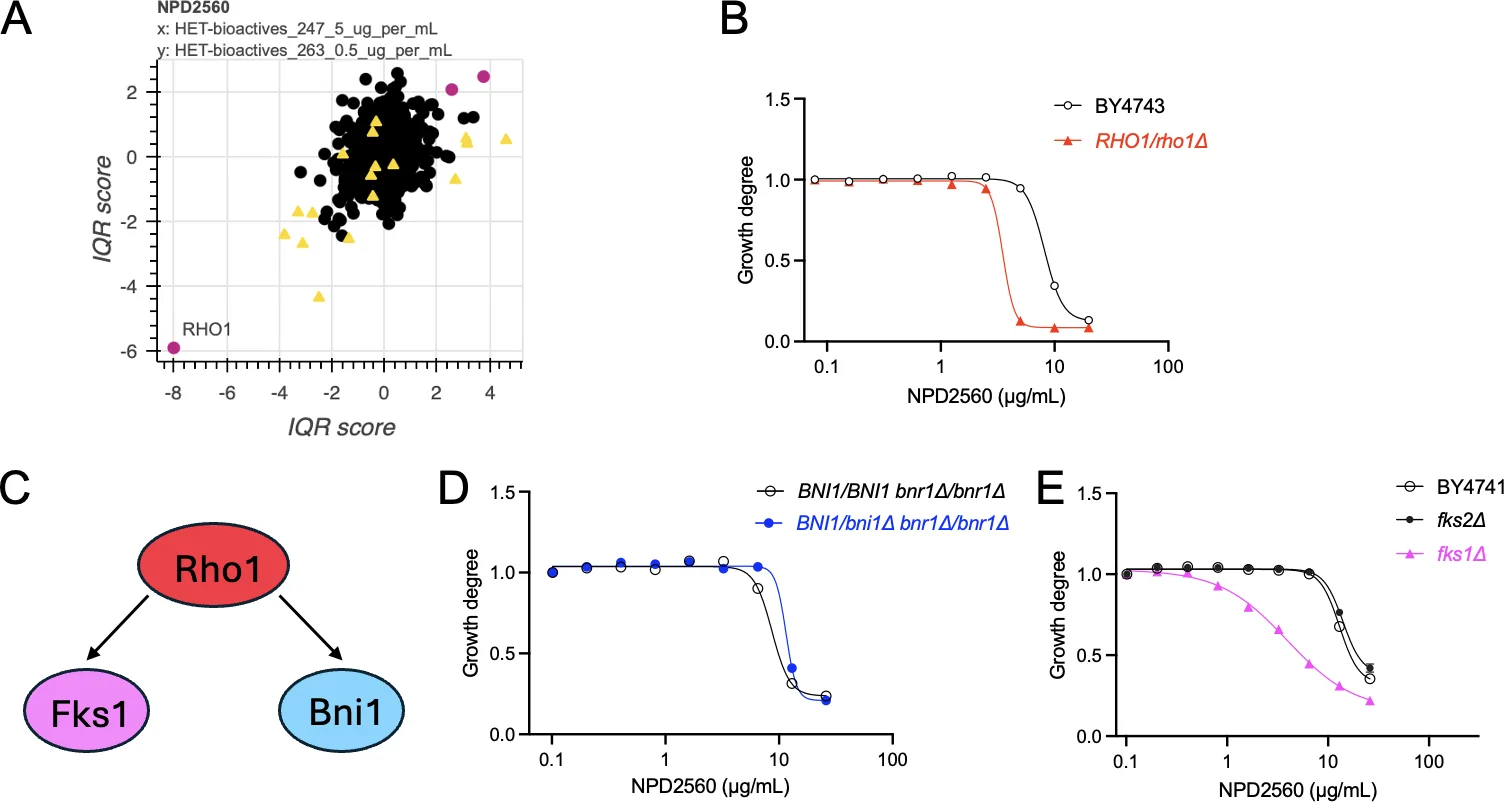
Chemical-genomic profiling and drug susceptibility testing of NPD2560. (**A**) Chemical-genetic interaction profile. Scatter plot of chemical-genetic interaction scores (IQR scores) obtained from pooled mutant screening. Each dot represents a heterozygous diploid deletion strain from the *S. cerevisiae* diagnostic mutant collection. The *x*- and *y*-axes show interaction scores for NPD2560 treatment at 5 and 0.5 μg/mL, respectively. Negative scores indicate hypersensitivity, whereas positive scores indicate resistance. Yellow triangles denote strains with reproducible differential sensitivity across concentrations. The *RHO1*/*rho1*Δ heterozygous mutant (magenta) exhibited a strong negative interaction. Chemical-genomic profiling was performed in the drug-hypersensitive Y13206 background. (**B**) NPD2560 sensitivity of the *RHO1*/*rho1*Δ strain. The WT strain used in this experiment was BY4743. (**C**) Schematic of the Rho1 signaling pathway, in which Rho1 regulates β-(1,3)-glucan synthesis via Fks1 and actin organization via Bni1. (**D**) NPD2560 sensitivity of the *BNI1*/*bni1*Δ bnr1Δ/*bnr1*Δ strain. (**E**) NPD2560 susceptibility assay. WT (BY4741), *fks2*Δ, and *fks1*Δ mutants were cultured in YPD medium containing the indicated concentrations of NPD2560 at 30°C for 18 h. Growth was quantified by OD_600_, normalized relative to OD_600_ = 1 under the control conditions (*n* = 3).

To identify the cellular pathways affected by NPD2560, we performed chemical-genomic profiling using a pooled collection of *S. cerevisiae* heterozygous deletion mutants collectively representing nearly all essential genes (23). Each mutant strain was exposed to NPD2560 or DMSO as a control, and barcode sequencing was performed to quantify changes in relative fitness across the population. Comprehensive fitness profiling revealed that the heterozygous deletion mutant *RHO1*/*rho1*Δ was among the strains most hypersensitive to NPD2560 (Fig. 2A). To validate this finding, we performed a drug susceptibility assay, which confirmed that *RHO1*/*rho1*Δ exhibited markedly increased sensitivity (IC_50_ = 3.51 µg/mL) compared with the parental control strain BY4743 (IC_50_ = 14.2 µg/mL) (Fig. 2B; Table 2). These results suggested that NPD2560 interferes with Rho1-dependent pathways involved in cell wall biogenesis.

**TABLE 2:** NPD2560 susceptibilities of yeast strains

Strain description	IC_50_ (μg/mL)	SE
BY4743	8.15	0.14
*RHO1*/*rho1*Δ	3.51	0.09
*BNI1*/*BNI1 bnr1*Δ/*bnr1*Δ	8.67	0.49
*BNI1*/*bni1*Δ *bnr1*Δ/*bnr1*Δ	11.09	1.25
BY4741	10.32	0.10
*fks2*Δ	14.03	0.79
*fks1*Δ	3.93	0.37

To clarify further the involvement of Rho1, we examined the drug sensitivity of mutants lacking Rho1 regulatory factors. As a small GTPase, Rho1 cycles between an inactive GDP-bound and an active GTP-bound state, with this transition controlled by guanine nucleotide exchange factors (GEFs) and GTPase-activating proteins (GAPs) (Fig. S1A). We tested strains with mutations in the Rho1 GEFs (*tus1*Δ, *rom1*Δ, and *rom2*Δ) and GAPs (*bem2*Δ, *bem3*Δ, *lrg1*Δ, *bag7*Δ, and *sac7*Δ). The drug susceptibility assay revealed that the *rom2*Δ mutant exhibited marked hypersensitivity to NPD2560 (IC_50_ = 0.08 µg/mL), whereas the *bem2*Δ mutant showed mild resistance (IC_50_ = 2.83 µg/mL) compared with the corresponding wild-type (WT) control (Fig. S1B; Table S2). These reciprocal phenotypes suggested that Rho1 activation status critically influences cellular sensitivity to NPD2560, with loss of the major activator Rom2 enhancing sensitivity and loss of the inactivator Bem2 partially alleviating sensitivity.

### NPD2560 perturbs the Rho1–Fks1 branch of the cell wall integrity pathway

Rho1 acts as a molecular switch and central regulator of cell proliferation, coordinating cell wall biogenesis and polarized growth (24). Among its essential downstream effectors, Fks1 serves as the catalytic subunit of β-(1,3)-glucan synthase (25), which directly drives glucan polymerization, whereas Bni1, a formin protein, promotes actin cable assembly and polarized growth (26). To determine which branch of the Rho1 pathway is most critical for cellular tolerance to NPD2560 (Fig. 2C), we compared drug sensitivity levels among mutants defective in these branches. The *fks1*Δ mutant exhibited pronounced hypersensitivity to NPD2560 with an IC_50_ of 3.93 μg/mL, whereas the *BNI1*/*bni1*Δ *bnr1*Δ/*bnr1*Δ diploid mutant did not show increased susceptibility compared to the control strain (Fig. 2D and E; Table 2). Notably, these assays were performed in the BY4743 background, distinct from the Y13206 background used in the previous section, and therefore IC₅₀ values are not directly comparable across figures. These results indicate that the integrity of the Rho1–Fks1 axis is particularly important for cellular tolerance to NPD2560, rather than implicating the Bni1-mediated actin polarity branch.

We next examined a panel of temperature-sensitive (TS) *rho1* mutants (27) to clarify how NPD2560 influences distinct functional branches of Rho1 signaling (Fig. S2A). These mutants, each carrying point mutations in conserved functional domains, were classified into two phenotypic groups. The *rho1A*-type mutants (*rho1-2* and *rho1-5*) are defective in activating the CWI pathway but retain partial Fks1 activity, whereas the *rho1B*-type mutants (*rho1-3*, *rho1-4*, *rho1-10*, and *rho1-11*) show severe defects in β-(1,3)-glucan synthase activation (Fig. S3A). Upon exposure to NPD2560, nearly all TS mutants exhibited increased sensitivity, with *rho1-3* (L60P) showing the most pronounced effect (IC_50_ = 1.68 µg/mL), compared with the WT strain (IC_50_ = 8.34 µg/mL) (Fig. S2B; Table S2). The *rho1-3* mutation lies within the switch II region (residues 60–71), a structural element critical for Fks1 interaction and β-(1,3)-glucan synthase activation (27). The heightened sensitivity of *rho1-3* to NPD2560 was therefore consistent with a functional dependency on the Rho1–Fks1 signaling axis for maintaining cell wall biosynthetic control.

### Morphological analysis supported involvement of the Rho1–Fks1 axis in cellular responses to NPD2560

Morphological characterization provided additional support for perturbation of the Rho1–Fks1 branch of the cell wall biosynthetic pathway. We compared the cellular morphology of TS *rho1* mutants with NPD2560-treated WT cells. Quantitative image analysis revealed that NPD2560-treated cells exhibited a modest but significant reduction in cell size compared with DMSO-treated controls (Fig. S3B and C). In addition to this size reduction, NPD2560-treated cells displayed increased roundness and a slightly widened bud neck, features that were also observed in *rho1B*-type mutants (*rho1-3* and *rho1-4*), which are defective in Fks1-dependent signaling (Fig. S3A and D). In contrast, *rho1A*-type mutants (*rho1-2* and *rho1-5*), which are impaired in CWI signaling, exhibited enlarged and more spherical cells (Fig. S3A and D). The morphological similarity between NPD2560-treated cells and *rho1B*-type mutants, together with the strong hypersensitivity of *rho1-3* and *fks1Δ* mutants, suggests that intact Rho1–Fks1-dependent processes are important for cellular tolerance to NPD2560.

### NPD2560 is associated with reduced β-(1,6)-glucan levels

**Fig 3:**
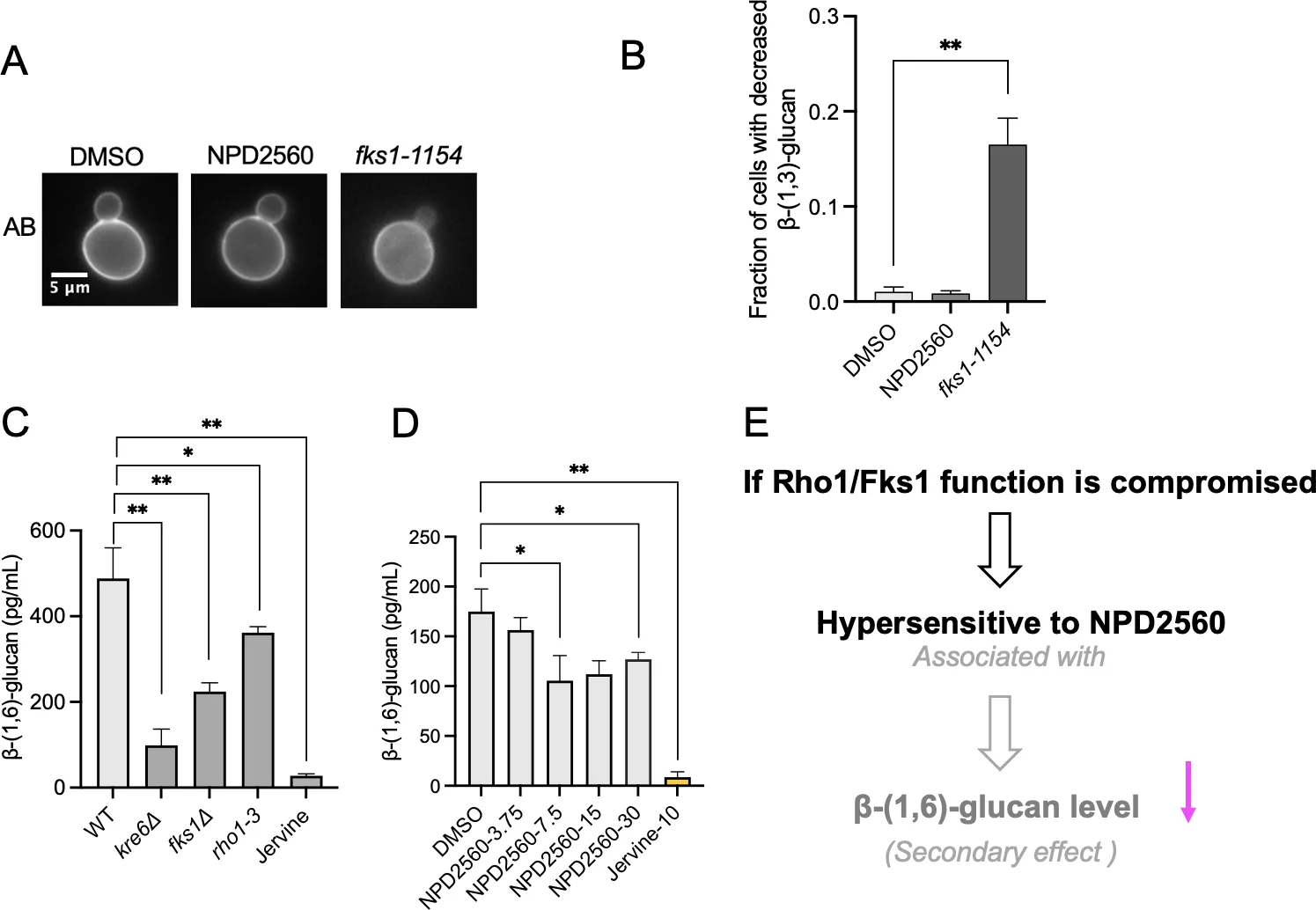
Effects of NPD2560 on β-(1,6)-glucan biosynthesis. (**A**) β-(1,3)-Glucan staining phenotype after NPD2560 treatment. Aniline blue staining of the WT (Y13206) and β-(1,3)-glucan synthase mutant *fks1-1154*. WT cells were grown to log phase in YPD at 30°C and stained after treatment with NPD2560. As *fks1-1154* is temperature-sensitive, it was grown overnight at 25°C and shifted to 37°C for 4 h. (**B**) Cells with reduced β-(1,3)-glucan in the bud. More than 100 budded cells were analyzed; the percentage of cells with reduced β-(1,3)-glucan in the bud are shown. Error bars indicate SD. Significant differences between WT and *fks1-1154* are indicated (**P < 0.01; one-way ANOVA). (**C**) β-(1,6)-Glucan levels in WT (BY4741), *kre6*Δ, *fks1*Δ, *rho1-3*, and jervine-treated cells. The amount of β-(1,6)-glucan per cell was quantified using pustulan as the standard. Error bars represent SD from three biological replicates. Significant differences relative to WT are indicated (*P < 0.05; **P < 0.01; *t*-test with FDR correction). (**D**) β-(1,6)-glucan levels in WT (Y13206), NPD2560-treated cells, and jervine-treated cells. The amount of β-(1,6)-glucan per cell was quantified using pustulan as the standard. WT cells were treated with NPD2560 (0, 3.75, 7.5, 15, or 30 μg/mL) or jervine (10 μg/mL). Error bars represent SD from three biological replicates. Significant differences relative to untreated WT (0 μg/mL) are shown (*P < 0.05; **P < 0.01; *t*-test with FDR correction). (**E**) Genetic dependency framework for NPD2560 sensitivity. This schematic summarizes genetic dependencies inferred from chemical-genomic and phenotypic analyses. Cells with compromised Rho1 or Fks1 function exhibit increased hypersensitivity to NPD2560, suggesting that the integrity of the Rho1–Fks1 regulatory network contributes to cellular tolerance to NPD2560-induced stress. Altered β-(1,6)-glucan levels are depicted as a secondary consequence associated with perturbation of this regulatory network. The model illustrates inferred functional relationships and does not imply direct molecular inhibition or a defined biochemical target of NPD2560.

*FKS1* encodes the catalytic subunit of β-(1,3)-glucan synthase (25) and has also been suggested as having a role in β-(1,6)-glucan biosynthesis (28). *RHO1* has also been implicated in both β-(1,3)-glucan and β-(1,6)-glucan biosynthesis (29, 30). To determine whether NPD2560 affects glucan biosynthesis, we first examined its impact on β-(1,3)-glucan production. WT cells were treated with NPD2560 and stained with aniline blue to visualize β-(1,3)-glucan. The fluorescence intensity and staining pattern of NPD2560-treated cells were indistinguishable from those of untreated controls and clearly different from the β-(1,3)-glucan synthase mutant *fks1-1154*, which showed markedly reduced fluorescence in the bud region (Fig. 3A and B). These observations indicate that under the conditions tested, NPD2560 did not significantly affect β-(1,3)-glucan synthesis. We next quantified β-(1,6)-glucan content by sandwich ELISA-like assay (31). Consistent with previous reports, both the *fks1*Δ mutant and TS *rho1-3* mutant exhibited markedly decreased β-(1,6)-glucan levels (Fig. 3C). Treatment with NPD2560 also led to a significant reduction in β-(1,6)-glucan content (*P*  <  0.05, Welch’s *t*-test with FDR correction), showing a <40% decrease at 7.5 μg/mL. Interestingly, further increases in NPD2560 concentration did not cause additional reductions. In contrast, jervine, which directly targets Kre6 and Skn1 involved in the β-(1,6)-glucan synthesis pathway, caused a >90% reduction at 10 μg/mL (Fig. 3D). To further investigate the underlying mechanisms, we examined the effects of NPD2560 on *kre6* and *skn1* mutants. The *kre6*Δ mutant showed slight hypersensitivity to NPD2560 (IC_50_ = 7.59 µg/mL), but the effect was much weaker than that observed with jervine. The *KRE6*(F552I) mutant, which exhibits resistance to jervine, due to reduced binding affinity (11), showed neither sensitivity nor resistance to NPD2560 (IC_50_ = 10.32 µg/mL) (Fig. S4; Table S2). These results suggested that, although NPD2560 decreased β-(1,6)-glucan levels, its mode of action differed from that of jervine and did not appear to involve direct inhibition of Kre6 or Skn1. Taken together, these observations indicate that cellular sensitivity to NPD2560 perturbs the Rho1–Fks1 pathway and is associated with reduced β-(1,6)-glucan levels under the conditions tested.

### NPD2560 triggers cell wall stress and activates the cell wall integrity pathway

**Fig 4:**
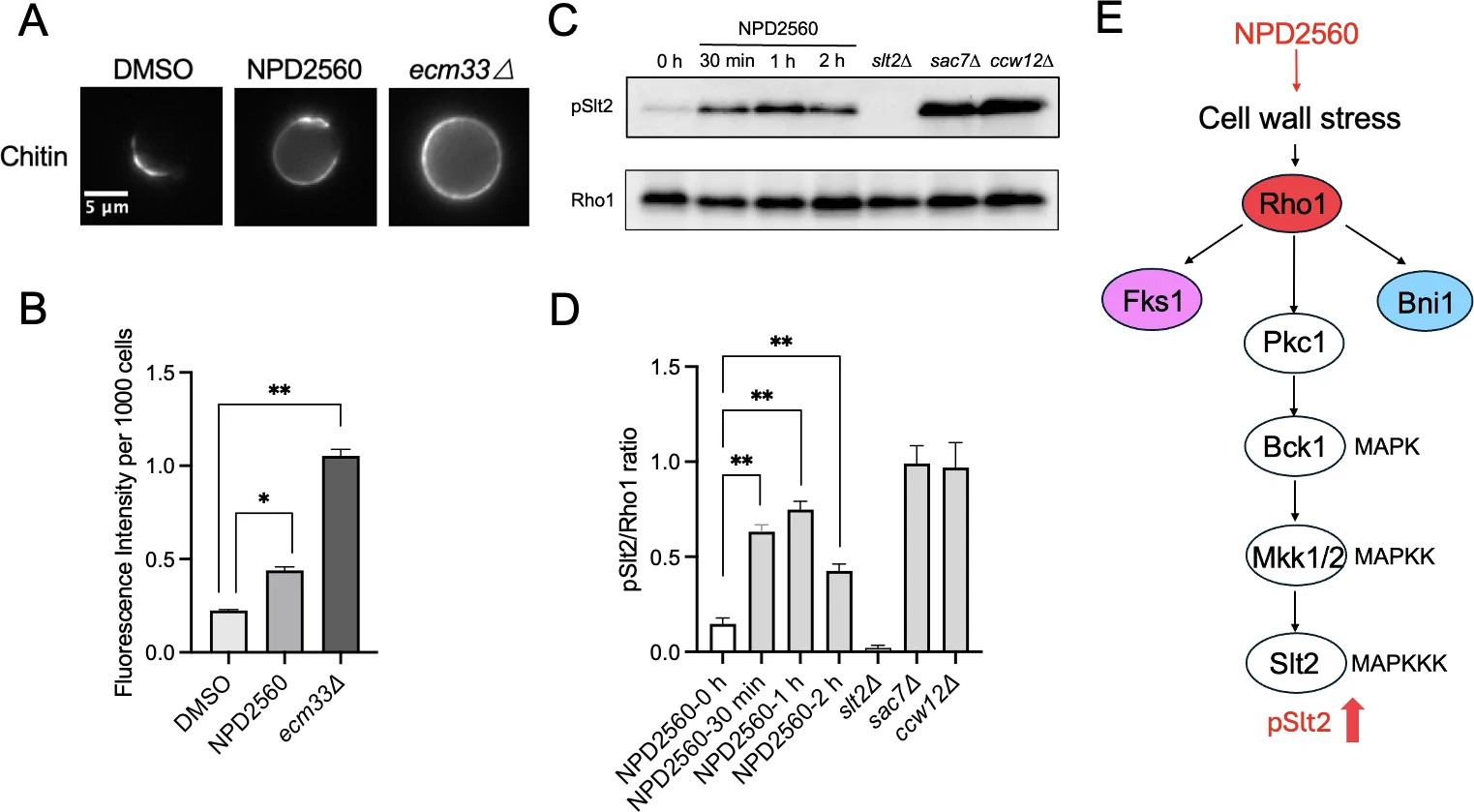
NPD2560 induces cell wall damage and activates the CWI pathway. (**A**) Chitin staining phenotype after NPD2560 treatment. WGA staining of WT (Y13206) and the chitin-deposition mutant *ecm33*Δ. (**B**) Quantification of chitin staining intensity. Error bars indicate SD. Significant differences between WT (Y13206) and NPD2560-treated cells or *ecm33*Δ are indicated (*P < 0.05; **P < 0.01; one-way ANOVA). (**C**) Western blotting analysis of phosphorylated Slt2 (pSlt2; upper panel) and Rho1 loading control (lower panel). Anti-phospho-p42/44 MAPK (T202/Y204) and anti-Rho1 antibodies were used to detect pSlt2 and Rho1, respectively. The WT (Y13206) cells were treated with NPD2560 and were collected at different hours. *slt2*Δ served as a negative control, and *sac7*Δ and *ccw12*Δ served as positive controls for pSlt2. (**D**) Quantification of pSlt2 levels from three independent experiments, presented as percentages relative to untreated WT (Y13206). Significant differences are indicated (*P < 0.05; **P < 0.01; one-way ANOVA). (**E**) Schematic of the CWI pathway, highlighting Slt2 as the MAP kinase activated in response to cell wall stress.

As NPD2560 treatment was associated with reduced β-(1,6)-glucan levels, we next examined whether it can also affect other components of the fungal cell wall. Fluorescent staining was performed using FITC-conjugated concanavalin A (FITC–ConA) for mannoproteins and wheat germ agglutinin (WGA) for chitin. NPD2560-treated cells showed normal mannoprotein staining, comparable to WT controls (Fig. S5A and B). In contrast, elevated levels of surface-exposed chitin detected by WGA were observed along the mother cell wall, resembling the phenotype of the *ecm33*Δ mutant (32) defective in cell wall organization (Fig. 4A and B). Consistent with these observations, Calcofluor White staining showed enhanced total chitin staining in NPD2560-treated cells (Fig. S5C). Quantification of Calcofluor White fluorescence intensity at the single-cell level further confirmed increased chitin accumulation upon NPD2560 treatment (Fig. S5D). Chitin accumulation is a well-established hallmark of cell wall damage, which is usually accompanied by activation of the CWI pathway mediated by the MAP kinase Slt2 (33). To confirm this activation, we analyzed Slt2 phosphorylation (pSlt2). Western blotting analysis revealed that NPD2560 treatment induced a strong but transient increase in pSlt2 activity, which was detectable at 30 min, peaked at 1 h, and diminished thereafter (Fig. 4C and D). For comparison, increased pSlt2 activity was also observed in the *sac7*Δ and *ccw12*Δ mutants, which served as positive controls known to exhibit constitutive CWI pathway activation due to cell wall defects (32). Taken together, these findings indicate that NPD2560 induces a cell wall stress response, leading to activation of the CWI MAPK cascade and compensatory chitin deposition. The observed cell wall stress response was consistent with functional perturbation of Rho1–Fks1-associated cell wall regulatory processes and activation of the CWI pathway (Fig. 4E).

### Transcriptomic remodeling revealed a coordinated cell wall stress response to NPD2560

**Fig 5:**
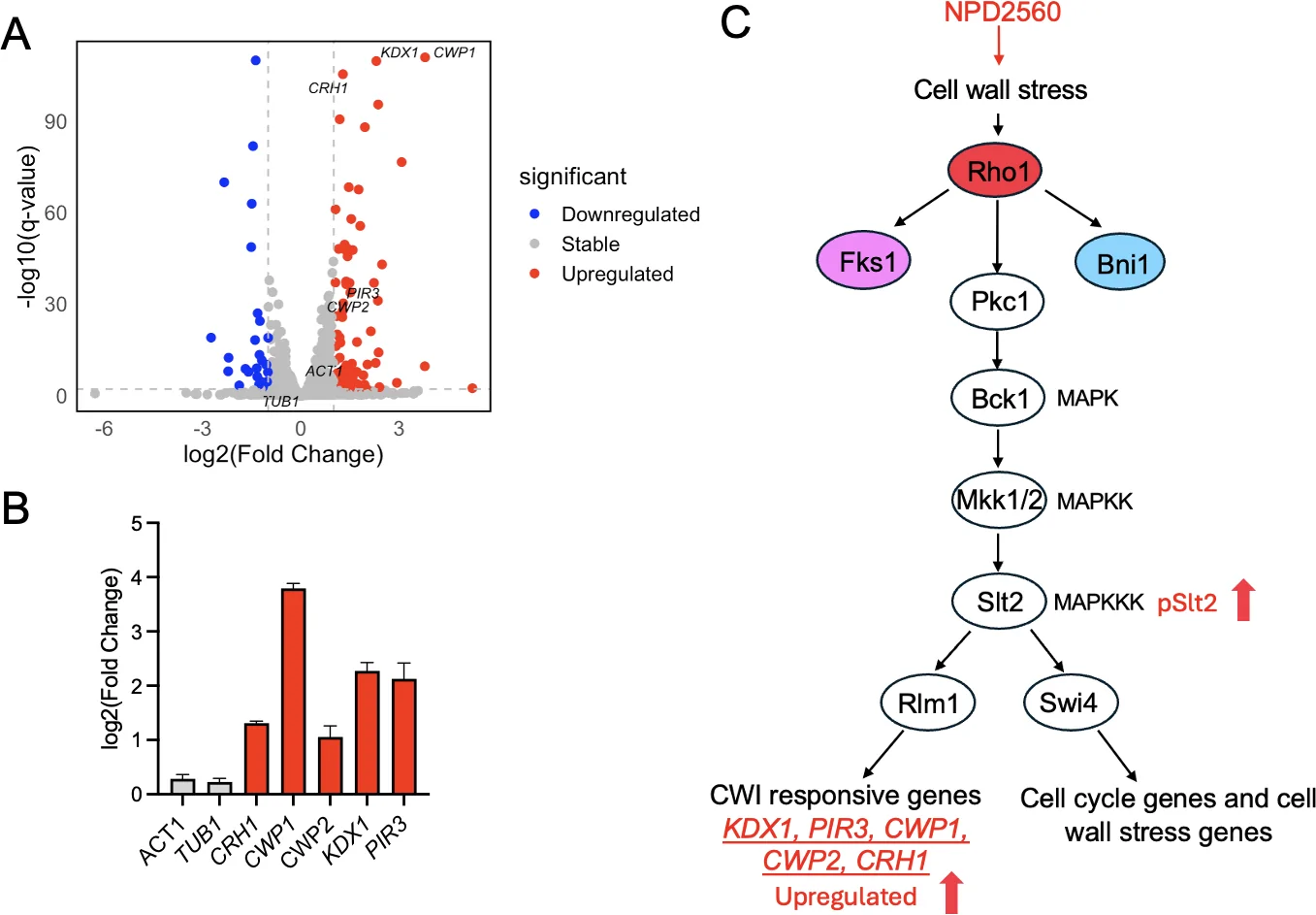
RNA-seq analysis after 2 h of NPD2560 treatment. (**A**) Volcano plot of differentially expressed genes (DEGs). Comparison of untreated (0 h) and NPD2560-treated (2 h) WT (BY4743) cells. Significantly up- and downregulated genes (*P*_adj_ < 0.01, |log_2_FC| > 1) are shown in red and blue, respectively; non-significant genes are in gray. Five cell wall stress-related genes (*CWP1*, *KDX1*, *CRH1*, *PIR3*, and *CWP2*) are highlighted, along with housekeeping controls (*TUB1* and *ACT1*). (**B**) RNA-seq-derived expression changes of selected CWI-responsive genes shown for clarity. Values are extracted from the RNA-seq data set shown in panel **A** and are presented to facilitate comparison of representative genes. Stress-related genes were strongly upregulated, while *TUB1* and *ACT1* expression remained stable. (**C**) Schematic of the CWI pathway highlighting transcriptional targets. Activation of Slt2 triggered downstream transcription factors, including SBF (Swi4–Swi6) and Rlm1. Five Rlm1-dependent, cell wall stress-responsive genes (*CWP1*, *KDX1*, *CRH1*, *PIR3*, and *CWP2*).

To capture the early transcriptional response following peak Slt2 activation but before detectable chitin accumulation, we performed RNA sequencing (RNA-seq) analysis of yeast cells treated with NPD2560 at the IC_80_-level concentration for 2 h. This time point allowed us to focus on the initial signaling phase of the CWI pathway. Correlation analysis confirmed high reproducibility among biological replicates (Pearson’s *r* = 0.983–0.997; Fig. S6). We identified 111 differentially expressed genes (DEGs; absolute log_2_FC > 1, adjusted *P* < 0.01), including 84 that were upregulated and 27 that were downregulated (Fig. 5A; Table S3). Gene ontology (GO) analysis showed significant enrichment in categories related to fungal-type cell wall and structural constituents of cell wall among the upregulated genes (Fig. S7A and B). Among the 30 most upregulated genes, 13 were directly associated with cell wall functions (Table S4), suggesting that NPD2560 treatment alters cell wall organization.

Activation of the CWI pathway, as evidenced by Slt2 phosphorylation, is known to promote transcription of stress response genes through the transcription factors Rlm1 and SBF (Swi4/Swi6 complex) (34). In agreement with this signaling model, RNA-seq data revealed coordinated upregulation of multiple Rlm1-regulated genes, including *CRH1*, *KDX1*, *CWP1*, *CWP2*, and *PIR3*, after NPD2560 treatment (Fig. 5B). These genes encode key enzymatic and structural components of the cell wall: *CRH1* encodes a chitin–β-(1,3)-glucan crosslinking enzyme; *KDX1* regulates β-(1,6)-glucan synthesis; *CWP1* and *CWP2* encode mannoproteins that stabilize the outer wall; and *PIR3* encodes a wall-anchored protein that contributed to crosslinking and mechanical strength. This transcriptional signature is consistent with a compensatory cell wall remodeling response (Fig. 5C). Taken together, these observations show that NPD2560 elicits a broad cell wall stress response characterized by activation of the CWI pathway and a transcriptional remodeling program that reinforces cell wall integrity.

## DISCUSSION

In this study, we investigated the cellular responses to NPD2560, a novel antifungal compound with fungicidal activity against *C. krusei*, using *S. cerevisiae* as a model system. Rather than fully elucidating a direct molecular target, genome-wide chemical-genomic profiling identified the small GTPase Rho1 as a key genetic node associated with hypersensitivity. Subsequent cell biological, biochemical, and transcriptomic analyses demonstrated that NPD2560 induces pronounced cell wall stress and functionally perturbs the Rho1-centered regulatory network, thereby leading to altered cell wall homeostasis, activation of the CWI pathway, and induction of Rlm1-dependent stress-responsive genes that together promote cell wall repair. These findings identify NPD2560 as a previously uncharacterized cell wall–active compound and provide insight into network-level regulation of fungal cell wall biogenesis.

### NPD2560 disrupts Rho1-dependent processes

Chemical-genomic profiling using the heterozygous gene deletion collection (HET) revealed that *RHO1*/*rho1*Δ was among the most hypersensitive strains to NPD2560, suggesting that intact Rho1 function is important for tolerance to NPD2560-induced stress. The validity of HET profiling for target identification has been well established, for example, in the identification of Erg11 as the primary target of fluconazole (35). However, heterozygous sensitivity can also reflect perturbation of essential pathways rather than direct target engagement. Given this reliability, we focused on Rho1, a small Rho-type GTPase that functions as an essential molecular switch coordinating cell wall biosynthesis, actin organization, and polarized growth (36).

Consistent with the HET results, TS *rho1* mutants exhibited hypersensitivity to NPD2560. Rho1 cycles between inactive GDP-bound and active GTP-bound states, a transition regulated by the GEF Rom2 and the GAP Bem2 (36, 37). Consistent with this regulation, the *rom2*Δ mutant showed strong hypersensitivity to NPD2560, whereas *bem2*Δ showed mild resistance, supporting the idea that reduced Rho1 signaling capacity exacerbates NPD2560 sensitivity. In contrast, the constitutively active *RHO1*(G19V) allele did not confer resistance (data not shown), suggesting that NPD2560 does not simply block Rho1 activation but instead perturbs downstream or parallel processes within the Rho1 regulatory network.

### NPD2560 perturbs the Rho1–Fks1–CWI network

Genetic analyses demonstrated that the Rho1–Fks1 branch of the cell wall regulatory network is particularly critical for cellular tolerance to NPD2560, rather than being conclusively identified as a direct molecular target. Perturbation of the Fks1-dependent branch would weaken cell wall integrity, which in turn would be sensed as wall stress resulting in activation of the CWI pathway composed of the Rho1–Pkc1–Slt2 MAPK cascade as a compensatory response. Consistent with this model, NPD2560 treatment induced a marked but transient increase in phosphorylated Slt2 (pSlt2), indicating activation of the CWI pathway. Moreover, the *bck1*Δ mutant, which is defective in the MAPKKK upstream of Slt2, exhibited hypersensitivity to NPD2560 (data not shown), supporting the view that CWI signaling becomes essential for survival following NPD2560-induced wall stress. Taken together, these results suggest that NPD2560 perturbs the Rho1–Fks1–CWI network, while not excluding the possibility of indirect mechanisms of action.

Unexpectedly, NPD2560 treatment was associated with a reduction in β-(1,6)-glucan levels, while β-(1,3)-glucan levels were largely unaffected under the conditions tested. Although both Rho1 (30) and Fks1 (28) have been implicated in β-(1,6)-glucan biosynthesis, the molecular basis for this relation has remained unclear. β-(1,6)-Glucan is covalently linked to β-(1,3)-glucan and contributes to the three-dimensional network that maintains cell wall integrity. Importantly, the modest sensitivity of *kre6*Δ cells and the lack of cross-resistance in the jervine-resistant *KRE6*(F552I) mutant argue against direct inhibition of β-(1,6)-glucan synthases. Instead, these findings are consistent with β-(1,6)-glucan reduction being a secondary consequence of perturbing Rho1–Fks1-dependent regulatory processes, rather than a primary target of NPD2560.

An alternative interpretation must also be considered. Hypersensitivity of *rho1* or *fks1*Δ cells may reflect an impaired capacity to mount adaptive CWI responses required to counteract NPD2560-induced cell wall stress, rather than direct inhibition of these proteins by the compound. In this scenario, NPD2560 would act primarily as a cell wall stressor, while intact Rho1–Pkc1–Slt2 signaling would be required to activate compensatory remodeling processes, including β-glucan and chitin synthesis. This network-level vulnerability model is well aligned with the genetic susceptibility profile observed in this study.

### Selectivity and safety implications of perturbing the Rho1 signaling pathway

Human RhoA is a functional homolog of yeast Rho1 and can complement the *rho1*Δ deletion in yeast (38), which raises potential concerns regarding cross-reactivity. However, Rho1 and RhoA share only ~72% amino acid identity, suggesting partial rather than complete conservation. In our analysis, NPD2560 sensitivity was comparable between *RHOA*/*RHOA* and *RHOA*/*rhoA*Δ cells in the *rho1*Δ/*rho1*Δ background, in marked contrast to the hypersensitivity observed in *RHO1*/*rho1*Δ strains (data not shown). This result argues against direct inhibition of RhoA by NPD2560. Nevertheless, comprehensive safety evaluation is an essential step in antifungal drug development, and further *in vivo* studies, such as mouse toxicity assays, will be required to confirm the biological specificity and potential therapeutic applicability of NPD2560.

### Implications of NPD2560 for antifungal research

Several coumarin derivatives, including coumarin itself, scopoletin, osthole, and umbelliferone derivatives, have been reported to exhibit antifungal activity against *Candida* species (39, 40). These compounds act primarily by inducing intracellular oxidative stress, disrupting mitochondrial function, or inhibiting biofilm formation (40, 41). In contrast, NPD2560 appears to perturb the regulatory architecture of fungal cell wall integrity, distinguishing it from previously characterized coumarin-based compounds.

Currently available antifungal drugs, including azoles, polyenes, and echinocandins, remain limited by toxicity, resistance, and narrow spectra of activity (42). Echinocandins, which inhibit β-(1,3)-glucan synthase, are ineffective against several NCAC species, such as *C. krusei* (43, 44). Although the antifungal activity of NPD2560 is currently limited in potency and spectrum, its ability to induce a defined cell wall stress response highlights its potential utility as a chemical probe for dissecting CWI network dependencies. Further optimization and mechanistic studies will be required to evaluate whether these properties can be translated into therapeutic potential.

## MATERIALS AND METHODS

### Strains

The drug-hypersensitive strain *S. cerevisiae* Y13206 (*3*Δ*; MATα pdr1*Δ*::natMX pdr3*Δ*::Kl.URA3 snq2*Δ*::Kl.LEU2 can1*Δ*::STE2pr-SP his5*Δ *his3*Δ *leu2*Δ *lyp1*Δ *met15*Δ *ura3*Δ) was derived from S288C. *S. cerevisiae his3*Δ (*MATa his3*Δ*::kanMX4 leu2*Δ *met15*Δ *ura3*Δ) strain is a derivative of BY4741 harboring a kanMX4 cassette at the *his3* locus. *S. cerevisiae* BY4743 (*MATa*/*α his3*Δ*1*/*his3*Δ*1 leu2*Δ*0*/*leu2*Δ*0 LYS2*/*lys2*Δ*0 met15*Δ*0*/*MET15 ura3*Δ*0*/*ura3*Δ*0*) is a diploid strain derived from S288C. The strains Y13206, *his3*Δ, and BY4743 were used as WT controls in their respective experimental contexts. Chemical-genomic profiling was performed in the drug-hypersensitive Y13206 background, as established in previous studies, to maximize sensitivity for detecting genetic interactions. Several targeted mutants analyzed in this study (e.g., *BNI1/bni1*Δ *bnr1*Δ*/bnr1*Δ) were newly constructed in the BY4743 background. RNA-seq analysis was performed using a wild-type, non-sensitized strain background to assess physiologically relevant transcriptional responses. *C. albicans* ATCC24433, *C. parapsilosis* ATCC6258, *C. tropicalis* ATCC750, and *C. krusei* ATCC22019 were obtained from the Medical Mycology Research Center, Chiba University. The strains used for the primary phenotypic and chemical-genomic analyses described in the main text are listed in Table 3, whereas the strains used exclusively for supplemental experiments are listed in Table S1.

**TABLE 3:** Fungal strains used in the main analyses in this study^*a*^

Species	Strain			Description
	YOC no.	Alias		
*Saccharomyces cerevisiae*	YOC5130	Y13206	*MATα pdr1*Δ::*natMX pdr3*Δ::*Kl.URA3 snq2*Δ::*Kl.LEU2 can1*Δ::*STE* *2pr-SP his5*Δ *his3*Δ *leu2*Δ *lyp1*Δ *met15*Δ *ura3*Δ	1*, 2*, 3*, 4*
		*his3*Δ	*MAT***a** *his3Δ*::*kanMX leu2*Δ *met15*Δ *ura3*Δ	1*
		BY4743	*MAT***a**/*MATα his3*Δ*1*/*his3*Δ*1 leu2*Δ*0*/*leu2*Δ*0 LYS2*/*lys2*Δ*0 met15*Δ*0*/*MET15 ura3*Δ*0*/*ura3*Δ*0*	1*, 5*
		*RHO1*/*rho1*Δ	*MAT***a**/*MATα his3*Δ*1*/*his3*Δ*1 leu2*Δ*0*/*leu2*Δ*0 LYS2*/*lys2*Δ*0 met15*Δ*0*/*MET15 ura3*Δ*0*/*ura3*Δ*0 RHO1*/*rho1*Δ::*kanMX*	1*
		*BNI1*/*BNI1 bnr1*Δ/*bnr1*Δ	*MAT***a**/*MATα his3*Δ*1*/*his3*Δ*1 leu2*Δ*0*/*leu2*Δ*0 LYS2*/*lys2*Δ*0 met15*Δ*0*/*MET15 ura3*Δ*0*/*ura3*Δ*0 bnr1*Δ/*bnr1*Δ	1*
		*BNI1*/*bni1*Δ *bnr1*Δ/*bnr1*Δ	*MAT***a**/*MATα his3*Δ*1*/*his3*Δ*1 leu2*Δ*0*/*leu2*Δ*0 LYS2*/*lys2*Δ*0 met15*Δ*0*/*MET15 ura3*Δ*0*/*ura3*Δ*0 bnr1*Δ/*bnr1*Δ *BNI1*/*bni1*Δ::*LEU2*	1*
	YOC4002	BY4741	*MAT***a** *his3*Δ *leu2*Δ *met15*Δ *ura3*Δ	1*, 2*
		*fks1*Δ	*fks1*Δ as BY4741	1*, 2*
		*kre6*Δ	*kre6*Δ as BY4741	2*
		*fks2*Δ	*fks2*Δ as BY4741	1*
	YOC784	*RHO1*	*MAT***a** *ade2*Δ *his3*Δ *leu2*Δ *lys2*Δ *trp1*Δ *ura3*Δ *rho1*Δ::*HIS3 ade3*Δ::*RHO*1::*LEU2*	1*
	YOC2435	*rho1-3*	*rho1-3* as *RHO1*	2*
		*sac7*Δ	*sac7*Δ as Y13206	1*, 4*
		*ecm33*Δ	*ecm33*Δ as Y13206	3*
		*mnn10*Δ	*mnn10*Δ as Y13206	3*
		*ccw12*Δ	*ccw12*Δ as Y13206	4*
		*slt2*Δ	*slt2*Δ as Y13206	4*
	YOC1087	*fks1-1154*	*MAT***a** *ade2Δ his3*Δ *leu2*Δ *lys2*Δ *trp1*Δ *ura3*Δ *fks1*Δ::*HIS3 fks2*Δ::*LYS2 ade3*Δ::*fks1-1154*:*TRP1*	3*
*Candida albicans*	YOC5265	ATCC24433		6*
*Candida parapsilosis*	YOC5268	ATCC22019		6*
*Candida tropicalis*	YOC5267	ATCC750		6*
*Candida krusei*	YOC5266	ATCC6258		6*

^*a*^ *1: Used in drug susceptibility tests, *2: Used in β-(1,6)-glucan quantification experiments, *3: Used in cell wall component staining experiments, *4: Used in western blotting analysis, *5: Used in RNA sequencing analysis, and *6: Used in resazurin cell viability assay.

### Media

Yeast cells were grown at 25°C or 30°C in yeast extract–peptone–dextrose (YPD)-rich medium containing 1% Bacto yeast extract (BD Biosciences, San Jose, CA, USA), 2% Bacto peptone (BD Biosciences), and 2% glucose (Fujifilm Wako Pure Chemical Corp., Osaka, Japan). *C. albicans* was grown at 35°C on Sabouraud agar medium containing 1% (wt/vol) Bacto-peptone (BD Biosciences), 4% (wt/vol) glucose (Fujifilm Wako Pure Chemical Corp.), and 1.5% (wt/vol) agar. For *Candida* susceptibility testing, RPMI 1640 (Fujifilm Wako Pure Chemical Corp.) was adjusted to pH 6.9 by addition of 165 mM 3-(*N*-morpholino) propanesulfonic acid (Fujifilm Wako Pure Chemical Corp.).

### Drugs

NPD2560 (STL514751; Vitas-M Laboratory, Moscow, Russia; Hong Kong, China), and jervine (J0009; Tokyo Chemical Industry, Tokyo, Japan) were purchased from the suppliers indicated. Jervine was used as a representative reference compound to contextualize genetic and phenotypic responses. All drugs were dissolved in dimethyl sulfoxide (DMSO; Fujifilm Wako Pure Chemical Corp.).

### Chemical-genomic profiling

Chemical-genomic profiling using the *S. cerevisiae* HET collection was performed as described previously (16, 17). Briefly, the barcoded HET library consisting of 968 diploid strains, each carrying a single-allele deletion of an essential gene, was pooled and cultured in YPGal medium (1% yeast extract, 2% peptone, and 2% galactose) at 30°C. Approximately 150 cells per strain were inoculated into 96-well plates in aliquots of 200 μL of culture per well and treated with serial concentrations of NPD2560 (0.125–32 μg/mL) in the presence of 1% DMSO. DMSO-only wells were used as untreated controls. After incubation for 24 h, the optical density at 600 nm (OD_600_) was recorded to estimate growth inhibition, and the cultures were further incubated for an additional 24 h before collection. The cells were harvested by centrifugation at 3,000 rpm for 4 min and treated with Zymolyase (0.5 mg/mL in 1 M d-sorbitol, 11.5 mM β-mercaptoethanol) for 1 h at 37°C. Genomic DNA was purified using a QIAamp 96 DNA kit (Qiagen, Hilden, Germany) on the QIAcube HT platform. Barcode sequences unique to each mutant were amplified by multiplex PCR using primers annealing to the kanMX cassette, resolved by agarose gel electrophoresis (2%), purified with a GENECLEAN III kit, and quantified by qPCR prior to Illumina HiSeq 2500 sequencing (RIKEN Center for Life Science Technologies, Yokohama, Japan). Sequencing reads were processed using the BEAN-counter pipeline (45). Strains with fewer than 20 barcode reads were excluded, and log-transformed read counts were normalized relative to the DMSO reference profile using LOWESS. Chemical-genetic interaction *z*-scores were calculated for each mutant strain, and outliers were identified based on Tukey’s interquartile range method. Profiles at concentrations showing the clearest separation between hypersensitive and resistant mutants were selected as representative data sets for further analysis.

### Antifungal susceptibility test using *S. cerevisiae* mutants

*S. cerevisiae* WT and mutant strains were cultured in YPD medium at 25°C or 30°C with shaking at 200 rpm until logarithmic growth (1 × 10^7^–5 × 10^7^ cells/mL). Overnight cultures were diluted with fresh YPD and inoculated into YPD containing 3% DMSO (with or without drug treatment) to an initial density of 1 × 10^5^–5 × 10^5^ cells/mL. Cultures were then incubated at 30°C under static conditions. NPD2560 was applied at concentrations ranging from 0 to 26 μg/mL. After 18-h incubation in 96-well flat-bottomed microtiter plates (Corning, Corning, NY, USA), cell suspensions were gently mixed using a Titramax 1000 rotator (Heidolph, Schwalbach, Germany), and the OD_600_ was measured with a SpectraMax Plus 384 plate reader (Molecular Devices, San Jose, CA, USA). The IC_50_ values were determined by fitting the dose–response data to a four-parameter logistic model using the drc package in R (46). To evaluate whether two IC_50_ estimates differed significantly, dose–response curves were additionally modeled with Markov chain Monte Carlo (MCMC) methods using the rstan package (https://mc-stan.org/users/interfaces/rstan), where the four-parameter log-logistic equation was reparameterized with the drc package (46). Statistical significance was assessed by likelihood ratio test comparing the full model (allowing all four parameters to vary among conditions) with a null model (allowing differences in all parameters except IC_50_). *P* values were determined from the chi-squared distribution and adjusted by the Bonferroni method. IC_50_ values for each strain are presented in Table 1 and Table S2.

### Resazurin cell viability assay

Susceptibility tests with *C. albicans* were performed using the Clinical and Laboratory Standards Institute document M60 (22). Briefly, *Candida* strains were grown on Sabouraud glucose agar for 24 h at 35°C, washed with 0.85% saline, and diluted in RPMI 1640 to 5 × 10^3^ cells/mL. Diluted cells and 3% DMSO (with/without drugs) were added to 96-well round-bottomed microplates and incubated at 35°C in a static incubator. NPD2560 concentrations ranged from 0 to 26 μg/mL. The fungal cell killing rate was determined by the resazurin cell viability assay as described previously (20). Living cells maintain a reducing environment within their cytoplasm and mitochondria, in which resazurin (blue and non-fluorescent) is reduced by dehydrogenase enzymes to form the red fluorescent dye resorufin. The amount of resorufin can be monitored by measuring fluorescence or absorbance, both of which are proportional to the number of living cells in the sample. Depending on the cell types, the resazurin assay can be used to detect as few as 40 cells reproducibly and with high sensitivity. After incubation of *C. albicans* ATCC24433 with drugs at various concentrations for 24 h, 10 μL (2.1 mg/mL) of resazurin was added to each well and incubated for 24 h. The color of each well was determined visually; a blue or purple color was interpreted as the absence of metabolic activity and dead cells, whereas pink indicated the presence of living fungal cells.

### Cell wall component staining

The WT strain Y13206 was cultured overnight at 30°C in YPD medium to logarithmic phase (1 × 10^7^–5 × 10^7^ cells/mL). The culture was then adjusted to 2 × 10^6^ cells/mL in YPD medium, and NPD2560 (2 μg/mL) was added at a final concentration of 0.03% DMSO. The cells were incubated with shaking at 30°C for 4 h. Aliquots of about 2 × 10^7^ cells were harvested at 0 and 4 h after treatment by centrifugation, washed twice with PBS, and subjected to mild sonication. Gene-deleted mutants (*mnn10*Δ and *ecm33*Δ) were cultured overnight at 30°C in YPD medium to logarithmic phase (1 × 10^7^–5 × 10^7^ cells/mL) and about 2 × 10^7^ cells were harvested. TS mutants (*fks1-1154*) were cultured overnight at 25°C in YPD medium to logarithmic phase (1 × 10^7^–5 × 10^7^ cells/mL), diluted to 2 × 10^6^ cells/mL, and incubated at the restrictive temperature (37°C) for 4 h. Staining of cell wall components was performed following the protocol described previously (47). For mannoprotein staining, the cells were stained with 1 mg/mL FITC–ConA (C7642; Sigma-Aldrich, St. Louis, MO, USA). For β-(1,3)-glucan staining, the cells were stained with 5 mg/mL aniline blue (016-21302; Fujifilm Wako Pure Chemicals Corp.). For chitin staining, the cells were stained with 5 mg/mL Tetramethylrhodamine-conjugated WGA (W849; Thermo Fisher Scientific, Waltham, MA, USA) and Calcofluor White M2R/Fluorescent Brightener 28 (F3543; Sigma-Aldrich). Finally, the signal intensity was measured using a microplate reader (Infinite 200 PRO plate reader; Tecan, Zurich, Switzerland). For morphological observation of TS *rho1* mutants, the WT *RHO1* strain (YOC784), *rho1A* group mutants (*rho1-2* and *rho1-5*), *rho1B* group mutants (*rho1-3* and *rho1-4*), and NPD2560-treated cells were stained with FITC–ConA. The WT strains were cultured to log phase in YPD medium at 25°C and stained after treatment with NPD2560 for 16 h. The TS *rho1* mutants were cultured overnight at 25°C and then shifted to the restrictive temperature (37°C) for 4 h before staining.

### Quantification of β-(1,6)-glucan

Quantification of β-(1,6)-glucan was performed in two independent experimental settings: (i) comparison of wild-type and mutant strains, and (ii) assessment of drug-induced changes in a drug-hypersensitive background. In both cases, β-(1,6)-glucan extraction and quantification were carried out using the same protocol described below.

For strain comparison experiments, wild-type cells (BY4741 background), together with the indicated mutant strains (*fks1*Δ, *kre6*Δ*,* and *rho1-3*), were cultivated in YPD medium at 30°C and 25°C (*rho1-3* is a temperature-sensitive strain) with agitation at 200 rpm until reaching a density of approximately 1 × 10⁷ cells/mL. Where indicated, wild-type cells were treated with jervine (10 μg/mL).

For drug treatment experiments, wild-type cells in the drug-hypersensitive Y13206 background were cultivated under the same conditions as BY4741 and treated with NPD2560 at concentrations ranging from 0 to 15 μg/mL or with jervine (10 μg/mL).

Following incubation, the cultures were centrifuged at 15,000 × *g* for 3 min, and the resulting supernatant was discarded. The cell pellet was washed once, resuspended in PBS to 1 × 10^6^ cells/mL, and subsequently autoclaved for 20 min. After a second centrifugation at 15,000 × *g* for 1 min, the pellet was subjected to polysaccharide extraction. Extraction of β-(1,6)-glucan was performed using a slightly modified version of the procedure described previously (10). Briefly, 500 μL of 10% trichloroacetic acid (TCA) was added to the sample and incubated on ice for 10 min. The mixture was centrifuged at 15,000 × *g* for 3 min, and the resulting pellet was washed twice with distilled water. The pellet was then suspended in 500 μL of 1 N NaOH and heated at 75°C for 1 h. After neutralization with 500 μL of 1 M HCl and 10 mM Tris-HCl (pH 7.0), the suspension was centrifuged again at 15,000 × *g* for 1 min, and the supernatant containing solubilized β-(1,6)-glucan was kept on ice.

Quantification of total β-(1,6)-glucan was carried out according to the method described previously (31). Briefly, 96-well white microplates were coated with Neg1-E321Q-His (2 μg/mL) and incubated overnight at 4°C. The wells were washed with PBS containing 0.05% Tween 20 (PBST) and blocked for 1 h using PBST supplemented with 1% bovine serum albumin (BPBST). After washing, diluted samples and standard β-(1,6)-glucan (pustulan; InvivoGen, San Diego, CA, USA) were added and incubated for 1 h at room temperature. Subsequently, biotin-labeled Neg1-E321Q-His (2 μg/mL) in BPBST was added to the washed wells and incubated for a further 1 h. The plates were washed again and treated with streptavidin–horseradish peroxidase (HRP) (R&D Systems, Minneapolis, MN, USA) for 20 min. After removing unbound reagents, SuperSignal ELISA Femto substrate (Thermo Fisher Scientific) was added, and luminescence was detected using a GloMax microplate reader (Promega, Madison, WI, USA).

### Western blotting analysis

Yeast cells (Y13206) were collected after treatment with 2 μg/mL NPD2560 for different times. Then, whole protein was extracted using the alkaline-TCA method. Briefly, cells were pelleted, treated with 1 mL of N/β solution (0.25 N NaOH, 1% β-mercaptoethanol), and incubated on ice for 10 min. Then, 100 μL of 100% trichloroacetic acid was added and incubated on ice for 10 min. The supernatant was discarded after centrifugation at 15,000 × *g* for 2 min at 4°C. After washing with 500 μL of 1 M Tris-HCl (pH 8.0), the cells were resuspended in sodium dodecyl sulfate (SDS) sample buffer including 62.5 mM Tris-HCl (pH 6.8), glycerol (10%), SDS (2%), β-mercaptoethanol (2%), and bromophenol blue (0.005%), incubated for 10 min at 100°C, and pelleted. The protein samples were then adjusted to the same concentration using the XL-Bradford method (APRO, KY-1030). Next, the supernatants were subjected to SDS-PAGE (8%; Bio-Rad, Bio-Rad Laboratories, Inc., Hercules, CA, USA). The membranes were blocked with 2% non-fat milk in Tris-buffered saline containing 0.1% Tween-20 for 30 min at room temperature, followed by incubation with primary antibody against phosphorylated p44/42 MAPK (Erk1/2) (Phospho-p44/42 MAPK (Erk1/2) (Thr202/Tyr204) Antibody, #9101, diluted 1:1000 (Cell Signaling Technology, Danvers, MA, USA) at 4°C overnight. This antibody detects p44 (Erk1) and p42 (Erk2) MAPK when phosphorylated either individually or dually at Thr202/Tyr204 for Erk1 (Thr185/Tyr187 for Erk2). After washing with TBST, the membranes were incubated with HRP-conjugated secondary anti-rabbit antibody at room temperature for 45 min. Finally, proteins were detected with an enhanced chemiluminescence system (ECL Plus; Amersham, Little Chalfont, Buckinghamshire, UK). Grayscale analysis was performed using ImageJ (National Institutes of Health, Bethesda, MD, USA).

### RNA sequencing analysis

Overnight cultures of the BY4743 strain were inoculated into 50 mL of YPD medium in 500 mL flasks at a starting density of 2.5 × 10^6^ cells/mL. The cultures were incubated at 30°C with shaking at 100 rpm until the cell density reached 1 × 10^7^ cells/mL (approximately 4 h). At this point, 1 mL of untreated cells was collected as the 0-h control sample. The cells were pelleted by centrifugation at 12,000 × *g* for 1 min, snap frozen in liquid nitrogen, and stored at −80°C. For treated samples, NPD2560 was dissolved in dimethyl sulfoxide (DMSO) and added to the culture at a final concentration of 16 μg/mL, corresponding to the IC_80_ determined for the BY4743 strain in preliminary assays. The final DMSO concentration was 0.03%. The cells were harvested after 2 h of treatment under the same conditions as the control. All samples were prepared in biological triplicate. The total RNA extraction, cDNA library preparation, paired-end sequencing, and preliminary data analysis were performed by BGI (Shenzhen, China). RNA integrity was assessed prior to sequencing to ensure high-quality RNA for library construction.

## Data Availability

The data sets used and/or analyzed during the current study are available from the corresponding author on reasonable request.

## AFTER EPUB

*[This article was published on 3 June 2026 with Hiroki Sekiguchi's name appearing incorrectly in the byline. The byline was updated in the current version, posted on 9 June 2026.]*

## SUPPLEMENTAL MATERIAL

Supplemental figures and tables spectrum.03630-25-s0001.pdf Figures S1 to S7 and Tables S1 to S4.

ASM does not own the copyrights to Supplemental Material that may be linked to, or accessed through, an article. The authors have granted ASM a non-exclusive, world-wide license to publish the Supplemental Material files. Please contact the corresponding author directly for reuse.
