# Supplementary material for: The novel antifungal agent NPD2560 perturbs the Rho1-centered signaling network to induce a cell wall integrity response
Source: Microbiol Spectr. 2026 Jun 3;14(7):e03630-25. doi: 10.1128/spectrum.03630-25 (PMC13340020; doi:10.1128/spectrum.03630-25)
Supplement: Supplemental figures and tables — Figures S1 to S7 and Tables S1 to S4. [file spectrum.03630-25-s0001.pdf]

Supplemental material

Fig.S1

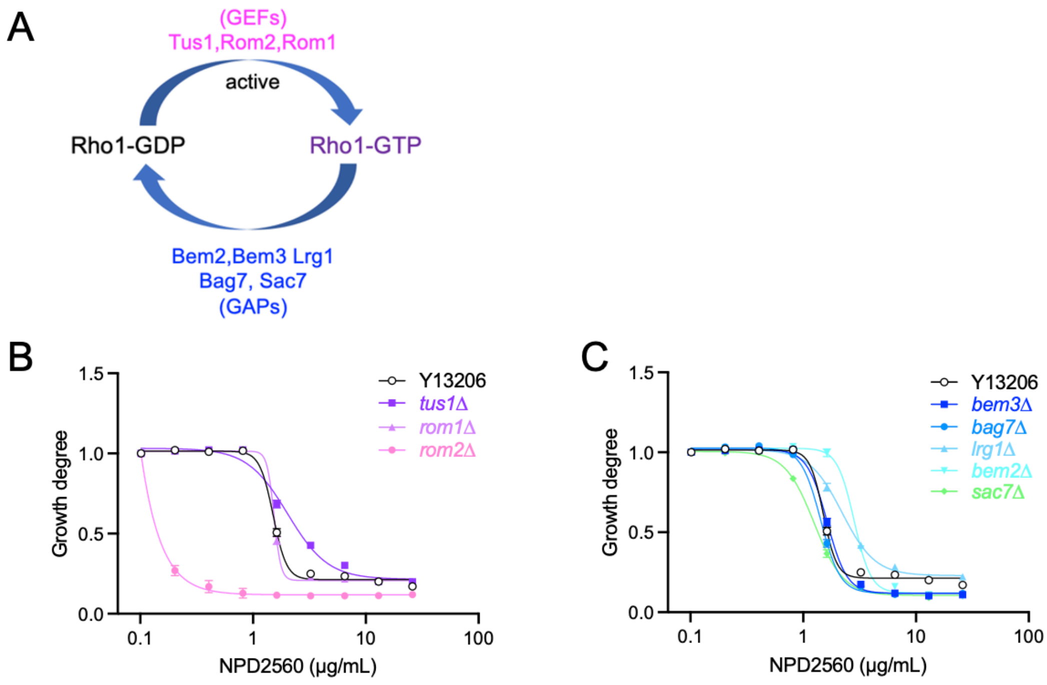

Supplementary Fig. S1. Drug susceptibility tests of Rho1's GAPs and GEFs mutants to NPD2560. (A) Schematic representation of the regulatory cycle of Rho1, a Rho-type GTPase. Rho1 is a kind of Rho-type GTPase, which is generally regulated by switching between a GDP-bound inactive state and a GTP-bound active state with conformational changes. The conversion between GTP- and GDP-bound form is regulated by GEFs and GAPs. (B) NPD2560 sensitivity of the mutants of GEFs. (C) NPD2560 sensitivity of the mutants of GAPs. Yeast mutant strains were incubated in YPD in the presence of the indicated concentrations of NPD2560 at 30°C for 18 h. The degree of proliferation was quantitated using the OD_{600}; $OD_{600} = 1$ in the control condition (n = 3).

Fig.S2

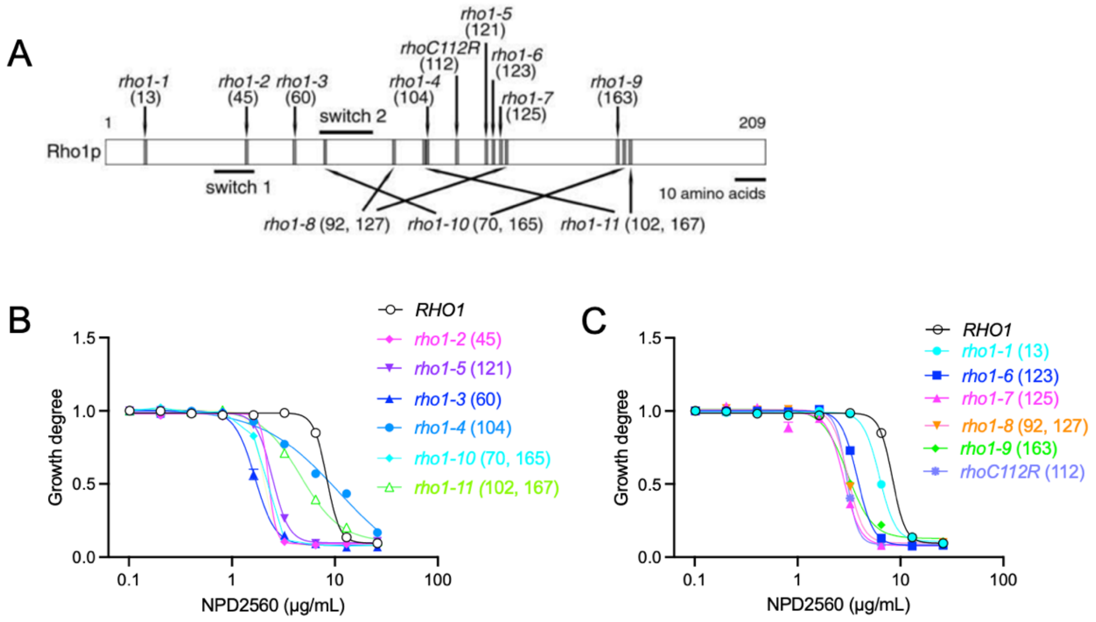

Supplementary Fig. S2. Drug susceptibility of *rho1* TS mutants to NPD2560. (A) Mutation sites in temperature-sensitive *rho1* mutants. Each of the 12 *rho1* mutants obtained has one or two mutated amino acids. Switch 1 (residues 33–49) and switch 2 (residues 60–71) constitute the putative effector recognition regions. Shaded bars indicate the mutation points. (B) NPD2560 sensitivity of *rho1* TS mutants. Because *rho1* TS mutants are temperature-sensitive mutant with very slow growth, the cells were cultured at 25°C in the presence of the indicated concentrations of NPD2560 for 40 h. The degree of proliferation was quantitated using the OD_{600}; OD_{600} = 1 in the control condition (n = 3).

Fig.S3

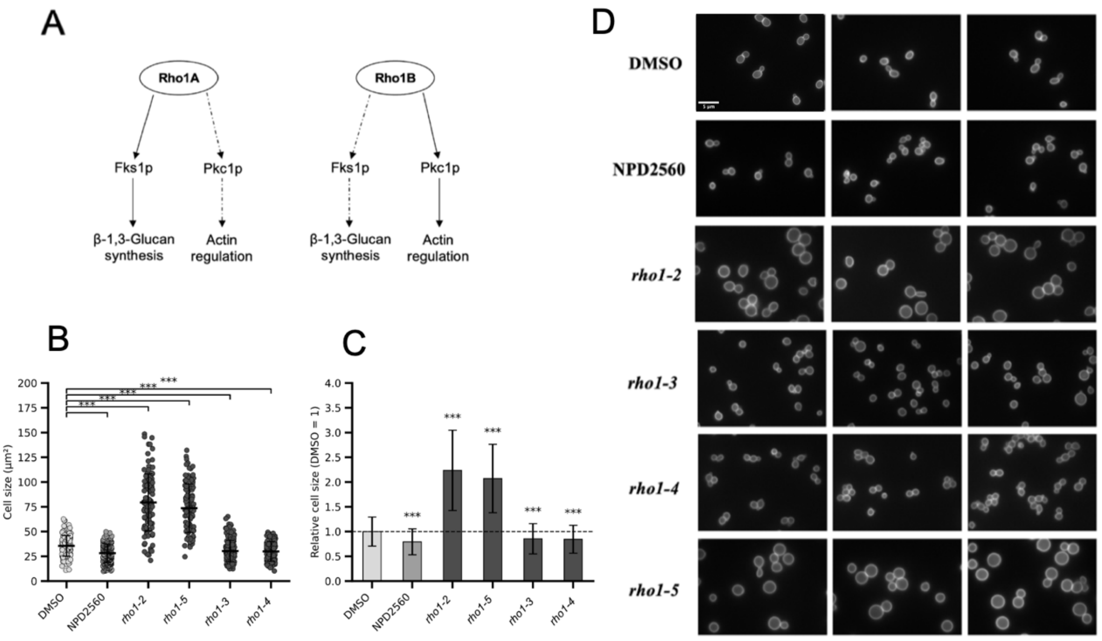

Supplementary Fig. S3. Morphology comparison between *rho1* TS mutants and NPD2560-treated cells. (A) Two *rho1* mutation groups (*rho1A* and *rho1B)* abolish the essential functions of Rho1. *rho1* mutants were categorized into two functional groups (24): the *rho1A* group (*rho1-2*, *rho1-5*), which lacks the ability to activate the Pkc1-Mpk1 pathway, and the *rho1B* group (*rho1-3*, *rho1-4*, *rho1-10*, *rho1-11*), which has severe defects in glucan synthase activation. Thin and thick arrows represent affected and unaffected control steps, respectively. (B) Cell size was quantified in individual cells. Data represent cells pooled from three independent experiments (N = 3). Each dot represents a single cell, and horizontal bars indicate the mean ± SD. Statistical significance was evaluated by Welch's t-test versus the DMSO control with Bonferroni correction for multiple comparisons (***, $P < 0.001$). (C) Relative cell size normalized to the mean value of the DMSO control. Bars indicate mean ± SD. (D) Morphology of rho1 TS mutants and NPD2560 treated cells. FITC-ConA staining of the wild type *RHO1* strain, *rho1A* group mutants (*rho1-2, rho1-5*), *rho1B* group mutants (*rho1-3, rho1-4*) and NPD2560 treated cells. The wild type strains were cultured to log phase in YPD medium at 25 °C and stained with NPD2560 after NPD2560 treatment for 16 h. Because the *rho1* mutants were temperature-sensitive mutant, cells were cultured overnight at 25°C and cultured for 4 h at restricted temperature (37°C).

Fig.S4

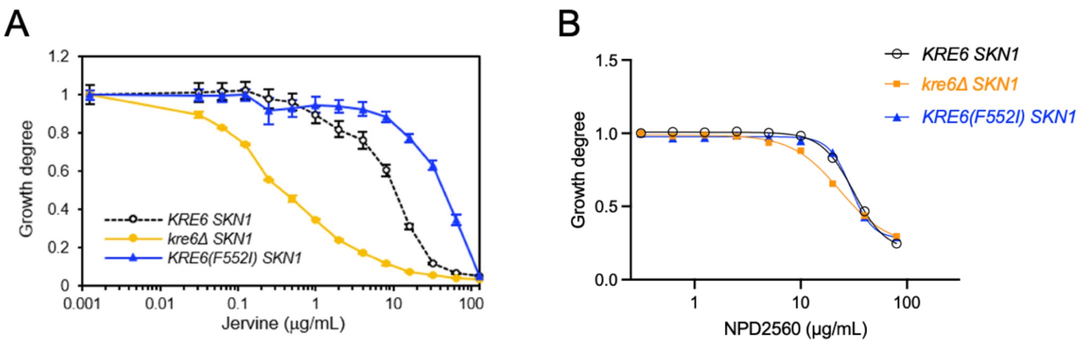

Supplementary Fig. S4. Drug susceptibility test of the *kre6* mutant to NPD2560. (A) Jervine susceptibility test (11). (B) NPD2560 susceptibility test. Wild type (*KRE6 SKN1*) and *kre6*Δ and point mutant *KRE6(F552I) SKN1* were cultured in YPD medium supplemented with the indicated concentrations of NPD2560 at 30°C for 18 h. The degree of proliferation was quantitated using the OD_{600}; OD_{600} = 1 in the control condition (n = 3).

Fig.S5

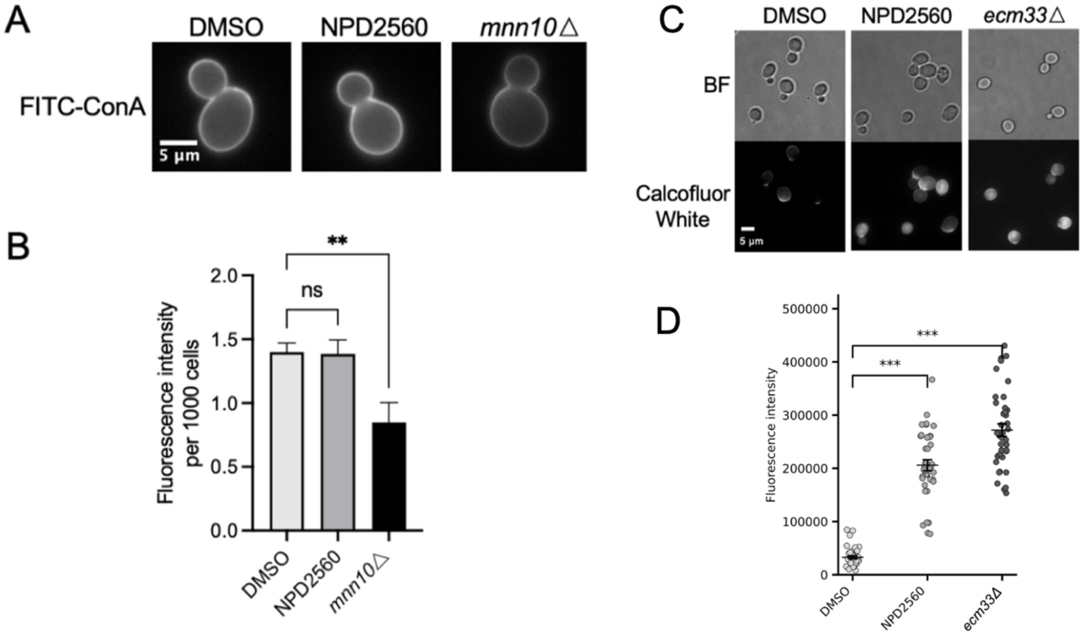

Supplementary Fig. S5. Mannoprotein staining phenotype after NPD2560 treatment. (A) FITC-ConA staining of the wild type, mannosyltransferase mutant (*mnn10*Δ). The yeast strains were cultured to log phase in YPD medium at 30°C and stained with FITC-ConA after NPD2560 treatment. (B) Fluorescence intensity of mannoprotein staining. Error bars indicate standard deviations. Significant differences between untreated wild-type cells and *mnn10*Δ mutant are indicated with asterisks (**, $P < 0.01$, after One-way ANOVA test). (C) Calcofluor White staining of WT (Y13206) and the chitin-deposition mutant *ecm33*Δ.WT cells were grown to logarithmic phase in YPD medium at 30 °C and stained with Calcofluor White after a 4-h treatment with NPD2560 or DMSO as a control. *ecm33*Δ cells were analyzed without drug treatment. (D) Increased fluorescence intensity in NPD2560-treated cells and the *ecm33*Δ mutant. Fluorescence intensity was quantified in individual cells. Data represent cells pooled from three independent experiments (N = 3; total n = 39 cells). Each dot represents a single cell, and horizontal bars indicate the mean ± SD. Cells treated with NPD2560 and the *ecm33*Δ mutant showed significantly higher fluorescence intensity than the DMSO control (Welch's t-test versus DMSO, ***, $P < 0.001$).

Fig.S6

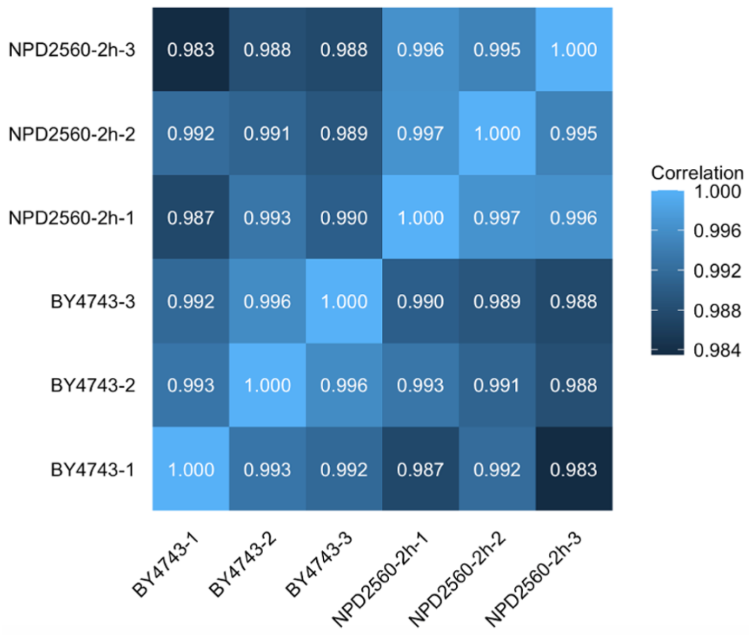

Supplementary Fig. S6. Sample Correlation Heatmap. The heatmap displays Pearson correlation coefficients between samples to assess experimental quality and reproducibility. Lighter shades indicate higher correlation values, with correlations nearing 1 observed between samples under the same treatment conditions (e.g., NPD2560-treated groups and untreated groups). This suggests highly consistent expression patterns within treatment groups and low technical variability.

Fig.S7

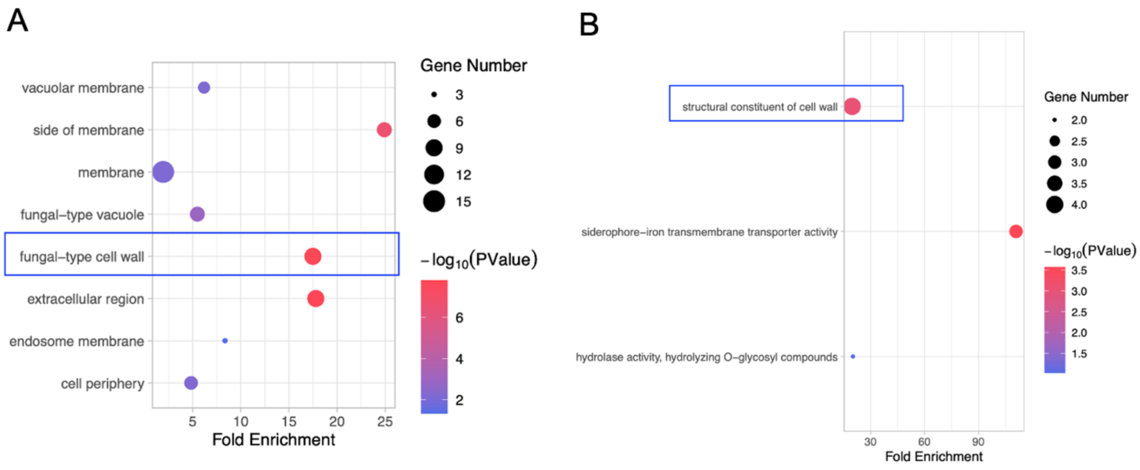

Supplementary Fig. S7. GO enrichment of top 30 up-regulated genes. The bubble plot displays the significantly enriched cell wall related GO terms in the (A) cellular component (CC) category and (B) molecular funcation (MF) category. The x-axis represents the fold enrichment, and the y-axis lists the enriched GO terms. The size of each bubble corresponds to the number of genes associated with the term, and the color indicates the -$\log_{10}$(P value). Lighter colors represent higher significance (greater -$\log_{10}$(P value)), while darker colors indicate lower significance.

TableS1 Fungal strains used for supplemental experiments.

Species	Strain		Genotype	Description
	**YOC No.**	**Alias**		
Saccharomyces cerevisiae	YOC5130	Y13206	*MATα pdr1Δ::natMX pdr3Δ::Kl.URA3 snq2Δ::Kl.LEU2 can1Δ::STE2pr-SP his5Δ his3Δ leu2Δ lyp1Δ met15Δ ura3Δ*	1*
		rom2Δ	*rom2Δ* as Y13206	1*
		rom1Δ	*rom1Δ* as Y13206	1*
		tus1Δ	*tus1Δ* as Y13206	1*
		bem2Δ	*bem2Δ* as Y13206	1*
		bem3Δ	*bem3Δ* as Y13206	1*
		lrg1Δ	*lrg1Δ* as Y13206	1*
		sac7Δ	*sac7Δ* as Y13206	1*
		bag7Δ	*bag7Δ* as Y13206	1*
	YOC4002	BY4741	*MATa his3Δ leu2Δ met15Δ ura3Δ*	1*
		kre6Δ SKN1	As BY4741 *kre6Δ::kanMX4*	1*
		KRE6 (F552I) *SKN 1*	As BY4741 *kre6Δ::URA3:KRE6(F552I):kanMX4*	1*
	YOC784	*RHO1*	*MAT***a** *ade2Δ his3Δ leu2Δ lys2Δ trp1Δ ura3Δ rho1Δ::HIS3 ade3Δ::RHO1::LEU2*	1*
	YOC771	*rho1-1*	*rho1-1* as YOC784	1*
	YOC772	*rho1-2*	*rho1-2* as YOC784	1*
	YOC2435	*rho1-3*	*rho1-3* as YOC784	1*
	YOC774	*rho1-4*	*rho1-4* as YOC784	1*
	YOC775	*rho1-5*	*rho1-5* as YOC784	1*
	YOC777	*rho1-6*	*rho1-6* as YOC784	1*
	YOC778	*rho1-7*	*rho1-7* as YOC784	1*
	YOC779	*rho1-8*	*rho1-8* as YOC784	1*
	YOC780	*rho1-9*	*rho1-9* as YOC784	1*
	YOC781	*rho1-10*	*rho1-10* as YOC784	1*
	YOC782	*rho1-11*	*rho1-11* as YOC784	1*
	YOC776	*rhoC112R*	*rhoC112R* as YOC784	1*
	YOC726	*RHO1(N46S)*	*MATα ade2Δ his3Δ leu2Δ lys2Δ trp1Δ ura3Δ rho1Δ::LYS2 ade3Δ::[pRHO1-rhoA:HIS3]*	1*
	YOC3003	*RHO1(G19V)*	*MATα ade2Δ his3Δ leu2Δ lys2Δ trp1Δ ura3Δ rho1Δ::HIS3 ade3Δ::[Rho1p-RHO1(G19V)]::LEU2*	1*

YOC3004	*RHO1(Q68L)*	*MATα ade2Δ his3Δ leu2Δ lys2Δ trp1Δ ura3Δ rho1Δ::LYS2 ade3Δ::[Rho1p-RHO1(Q68L)]::LEU2*	1*

*1: Used in drug susceptibility tests

TableS2 NPD2560 susceptibilities of yeast strains

Strain description	IC_{50} (μg/mL)	SE
Y13206	1.50	0.08
rom2Δ	0.08	0.00
rom1Δ	1.50	0.18
tus1Δ	2.03	0.21
bem2Δ	2.83	0.09
bem3Δ	1.63	0.03
lrg1Δ	2.21	0.09
BY4741	10.32	0.10
kre6Δ SKN1	7.59	0.45
KRE6 (F552I) SKN1	9.60	0.31
YOC784 (*RHO1,* parent)	8.34	9.23
rho1-1	6.24	0.08
rho1-2	2.27	0.15
rho1-3	1.68	0.04
rho1-4	10.64	4.36
rho1-5	2.48	0.05
rho1-6	3.81	0.05
rho1-7	2.81	0.20
rho1-8	3.08	0.02
rho1-9	3.05	0.10
rho1-10	2.02	0.04
rho1-11	4.65	0.19
rhoC112R	3.00	0.04
RHO1(N46S)	1.40	0.15
RHO1(G19V)	3.32	0.13
RHO1(Q68L)	3.61	0.07

TableS3 Differentially expressed genes (DEGs) identified at 2 h post NPD2560 treatment

Gene_id	Gene_symbol	log_2(Fold Change)	q-value	p-value
852875	*LEU1*	-1.3791591	1.32E-110	4.56E-114
852390	*PHO5*	-1.4613749	1.75E-82	2.42E-85
854146	*YOL014W*	-2.3397991	1.24E-70	2.14E-73
851299	*PHO11*	-1.4993299	1.37E-63	3.08E-66
852290	*RCR1*	-1.5163552	2.35E-49	7.31E-52
853751	*HAP4*	-1.3203135	1.25E-27	9.73E-30
854395	*RCN2*	-1.2520844	4.39E-25	4.09E-27
854567	*YOR385W*	-2.7411441	1.20E-19	1.47E-21
853203	*BIO2*	-1.0012454	1.27E-19	1.58E-21
852252	*YBL029W*	-1.398337	7.34E-19	9.62E-21
851613	*NRG1*	-1.2636353	5.17E-14	9.26E-16
854144	*CMK2*	-2.2072845	4.68E-13	9.03E-15
851356	*YDL241W*	-1.1929854	3.19E-12	6.88E-14
852641	*HFM1*	-1.0641963	8.86E-11	2.17E-12
856540	*YHR138C*	-1.0557626	9.33E-11	2.32E-12
853043	*BTN2*	-1.3489069	1.38E-09	3.81E-11
850743	*OSW2*	-1.6923327	2.06E-09	5.80E-11
1466443	*MNC1*	-2.2195519	1.37E-08	4.23E-10
855364	*DIA1*	-1.5983727	2.10E-08	6.69E-10
852358	*NRG2*	-1.0093807	2.49E-08	8.20E-10
850364	*YCR007C*	-1.3310139	7.98E-07	3.43E-08
851512	*STP4*	-1.2235746	3.05E-05	1.83E-06
854008	*IMA2*	-1.0325875	3.79E-05	2.33E-06
855167	*GAT2*	-1.277769	0.00031742	2.59E-05
850385	*HSP30*	-1.8813892	0.0006102	5.44E-05
850959	*YLR256W-A*	-1.1560369	0.00172405	0.00018388
856349	*MUP3*	-1.2042807	0.00967316	0.0013826
853766	*CWP1*	3.7888897	0	0
853696	*KDX1*	2.30173635	2.14E-110	1.11E-113

853102	*CRH1*	1.2824451	4.42E-106	3.05E-109
856616	*CRG1*	2.35926531	3.77E-96	3.25E-99
853757	*GFA1*	1.18347325	2.73E-91	2.82E-94
856017	*YPL088W*	1.95286488	1.06E-88	1.28E-91
851987	*ARO10*	3.07466259	3.03E-77	4.70E-80
854789	*YKE4*	1.4615223	5.26E-69	9.98E-72
851649	*SED1*	1.76175067	3.18E-68	6.57E-71
851007	*EXG1*	1.05624842	9.66E-62	2.33E-64
856345	*ARN1*	1.53573026	1.40E-58	3.62E-61
854564	*FIT2*	1.810192	2.66E-56	7.35E-59
856350	*VMR1*	1.33411667	3.63E-50	1.06E-52
854556	*ALD4*	1.39563538	3.98E-49	1.30E-51
854010	*HPF1*	1.15407224	9.50E-49	3.28E-51
855667	*YNL058C*	1.34326978	1.45E-48	5.23E-51
856644	*SIT1*	1.58236803	2.13E-48	8.06E-51
856338	*ARN2*	1.42339238	2.49E-46	9.87E-49
852147	*FIT1*	2.47647659	1.15E-43	4.95E-46
852179	*BNA4*	1.37885184	4.53E-38	2.19E-40
854483	*MCH5*	1.05387323	1.01E-37	5.06E-40
853693	*PIR3*	2.23009748	1.32E-37	6.82E-40
854096	*ARG1*	1.48424896	1.34E-37	7.16E-40
850620	*UBI4*	1.38444655	3.79E-37	2.09E-39
855692	*NCE103*	1.50902575	1.96E-34	1.15E-36
850614	*HXK1*	2.34981069	9.07E-32	5.79E-34
850827	*TIS11*	1.29276691	6.73E-31	4.41E-33
853935	*KTR2*	1.27404726	2.08E-28	1.58E-30
853765	*CWP2*	1.09844147	1.02E-26	8.43E-29
850308	*YCL049C*	1.26472257	2.21E-26	1.90E-28
853357	*ARG3*	2.13363309	1.06E-21	1.10E-23
855222	*ADD37*	1.11145264	1.07E-20	1.23E-22
851862	*CCC2*	1.18144867	1.00E-19	1.21E-21
854565	*FIT3*	1.71091283	3.20E-18	4.25E-20

855562	*YGP1*	1.20718301	5.12E-18	6.97E-20
856791	*GPP2*	1.06976122	7.20E-17	1.05E-18
852934	*NQM1*	2.37051353	8.95E-15	1.56E-16
853912	*GAP1*	1.18537211	4.72E-13	9.20E-15
851802	*ADR1*	1.0536653	4.69E-12	1.03E-13
853966	*SRL3*	2.28062739	2.38E-11	5.42E-13
856289	*GPH1*	1.55853539	4.30E-11	1.02E-12
855484	*YTP1*	2.0304016	7.85E-11	1.89E-12
851238	*BDH2*	1.38158764	1.45E-10	3.64E-12
852721	*AMS1*	1.53033599	1.97E-10	5.04E-12
850532	*HSP12*	3.7808182	2.99E-10	7.74E-12
854788	*YIL024C*	1.51102826	1.68E-09	4.70E-11
850902	*HMX1*	1.31253031	2.33E-09	6.60E-11
854566	*FRE5*	1.34261793	9.07E-09	2.77E-10
855129	*YMR103C*	1.7097105	2.24E-08	7.22E-10
854857	*YPS6*	1.39730114	2.28E-08	7.39E-10
851056	*GAS2*	1.11730347	5.11E-08	1.78E-09
853003	*CLB6*	1.35896653	5.75E-08	2.04E-09
850331	*HBN1*	1.35944453	1.80E-07	6.84E-09
852691	*STR3*	1.9050539	2.67E-07	1.04E-08
851448	*TMA17*	1.00116592	3.25E-07	1.28E-08
1466465	*DPI8*	1.58021896	2.12E-06	9.75E-08
856324	*OPT2*	1.24786548	3.60E-06	1.73E-07
850972	*BOP2*	1.51412909	4.18E-06	2.04E-07
853951	*ECM4*	1.08553042	6.20E-06	3.18E-07
851943	*HXT7*	1.08948033	7.40E-06	3.85E-07
851304	*FMP45*	1.2789177	1.53E-05	8.62E-07
855292	*GTO3*	1.07406237	4.11E-05	2.57E-06
853541	*BNA2*	1.50446377	5.09E-05	3.28E-06
855451	*ALP1*	1.48834641	5.97E-05	3.91E-06
855120	*SNO1*	2.92809221	7.97E-05	5.55E-06
854416	*SSP2*	1.00424374	0.0001041	7.45E-06

852970	*YGR079W*	1.38228143	0.00015671	1.16E-05
854706	*XBP1*	1.73212084	0.00023012	1.79E-05
854834	*MET28*	1.52748551	0.00036173	3.02E-05
852978	*PDC6*	1.94535504	0.00036387	3.05E-05
852364	*HSP26*	1.80146689	0.00092999	8.83E-05
853792	*OSI1*	1.35703936	0.0009597	9.22E-05
854846	*DAL4*	1.60358524	0.00111902	0.00010939
853140	*AMA1*	1.6785803	0.0019472	0.00021184
856193	*YPR078C*	2.39679092	0.00207816	0.00022788
853007	*CLD1*	1.19508495	0.00220417	0.00024284
855518	*SPS19*	1.50417931	0.00310423	0.00036127
854859	*PAU15*	5.2344218	0.00474449	0.0005947
855130	*YPK2*	1.36335921	0.00552474	0.0007125
856496	*HXT5*	1.34010539	0.0059707	0.00078546
853279	*FMP33*	1.29603293	0.00611243	0.00080832
855527	*YNL194C*	1.77996296	0.00663332	0.00088864
850476	*YFL068W*	1.4808047	0.00852904	0.00119112
853158	*MPC3*	2.00860494	0.00864655	0.0012135

TableS4 Genes related cell wall among the top 30 up-regulated genes

Gene_id	Gene_symbol	log_2(Fold Change)	q-value	Description
853766	*CWP1*	3.7888897	0	Cell wall mannoprotein that localizes to birth scars of daughter cells; linked to beta-1,3- and beta-1,6-glucan heteropolymer through phosphodiester bond
853696	*KDX1*	2.30173635	2.14E-110	Protein kinase; implicated in Slt2p mitogen-activated (MAP) kinase signaling pathway; interacts with numerous components in the mating pheromone and CWI MAPK pathways
853102	*CRH1*	1.2824451	4.42E-106	Chitin transglycosylase; functions in the transfer of chitin to beta (1-6) and beta(1-3) glucans in the cell wall; similar and functionally redundant to Utr2; localizes to sites of polarized growth; expression induced by cell wall stress
856616	*CRG1*	2.35926531	3.77E-96	S-AdoMet-dependent methyltransferase involved in lipid homeostasis
853757	*GFA1*	1.18347325	2.73E-91	Glutamine-fructose-6-phosphate transaminase involved in cell wall chitin biosynthesis
851649	*SED1*	1.76175067	3.18E-68	Major stress-induced structural GPI-cell wall glycoprotein;
851007	*EXG1*	1.05624842	9.66E-62	Major exo-1,3-beta-glucanase of the cell wall; involved in cell wall beta-glucan assembly;
854564	*FIT2*	1.810192	2.66E-56	Mannoprotein that is incorporated into the cell wall; incorporated via a glycosylphosphatidylinositol (GPI) anchor; involved in the retention of siderophore-iron in the cell wall
854010	*HPF1*	1.15407224	9.50E-49	Putative glucosidase implicated in cell wall organization
852147	*FIT1*	2.47647659	1.15E-43	Mannoprotein that is incorporated into the

				cell wall; incorporated via a glycosylphosphatidylinositol (GPI) anchor; involved in the retention of siderophore-iron in the cell wall
853693	*PIR3*	2.23009748	1.32E-37	O-glycosylated covalently bound cell wall protein; required for cell wall stability; expression is cell cycle regulated, peaking in M/G1 and also subject to regulation by the cell integrity pathway;
853935	*KTR2*	1.27404726	2.08E-28	Golgi protein with mannosyltransferase activity; plays roles in biosynthesis of cell wall mannoproteins and N-linked protein glycosylation
853765	*CWP2*	1.09844147	1.02E-26	Covalently linked cell wall mannoprotein; major constituent of the cell wall; plays a role in stabilizing the cell wall;
